# Healthy ageing: the natural consequences of good nutrition—a conference report

**DOI:** 10.1007/s00394-018-1723-0

**Published:** 2018-05-24

**Authors:** D. Marsman, D. W. Belsky, D. Gregori, M. A. Johnson, T. Low Dog, S. Meydani, S. Pigat, R. Sadana, A. Shao, J. C. Griffiths

**Affiliations:** 1 Procter & Gamble, Cincinnati, OH USA; 2 Duke University, Raleigh-Durham, NC USA; 3 University of Padua, Padua, Italy; 4 University of Georgia, Athens, GA USA; 5 Integrative Medicine Concepts, Tucson, AZ USA; 6 Tufts University, Boston, MA USA; 7 Creme Global, Dublin, Ireland; 8 World Health Organization, Geneva, Switzerland; 9 Amway/Nutrilite, Buena Park, CA USA; 10 Council for Responsible Nutrition-International, Washington, DC USA

**Keywords:** Ageing, Biomarkers, Centenarians, Functional ability, Geroprotectors, Immunosenescence, Intrinsic capacity, Lifespan, Micronutrients, Minerals, Nutrition, Quality of life, Vitamins

## Abstract

Many countries are witnessing a marked increase in longevity and with this increased lifespan and the desire for healthy ageing, many, however, suffer from the opposite including mental and physical deterioration, lost productivity and quality of life, and increased medical costs. While adequate nutrition is fundamental for good health, it remains unclear what impact various dietary interventions may have on prolonging good quality of life. Studies which span age, geography and income all suggest that access to quality foods, host immunity and response to inflammation/infections, impaired senses (i.e., sight, taste, smell) or mobility are all factors which can limit intake or increase the body’s need for specific micronutrients. New clinical studies of healthy ageing are needed and quantitative biomarkers are an essential component, particularly tools which can measure improvements in physiological integrity throughout life, thought to be a primary contributor to a long and productive life (a healthy “lifespan”). A framework for progress has recently been proposed in a WHO report which takes a broad, person-centered focus on healthy ageing, emphasizing the need to better understand an individual’s intrinsic capacity, their functional abilities at various life stages, and the impact by mental, and physical health, and the environments they inhabit.

## From biology to quality of life: defining ‘healthy ageing’

Advances in science and medicine have led to increased life expectancy in high-income countries. By 2050, more than one-third of the population in high-income countries is expected to be of age 60 or older [144]. As a leading risk factor for chronic disease, ageing is also associated with reduced productivity and rising healthcare costs; age-related public spending is expected to double by 2050 in many countries [158]. This combination of disease and healthcare burden has piqued societies’ interest in the topic of ageing in recent years and led to research and medical initiatives aimed at slowing, delaying or even reversing the ageing process. To cope with these changes, the goal for societies should be to optimize intrinsic capacity and functional ability of individuals and populations as healthy ageing does not require people to be disease-free [154].

**Fig. 1:**
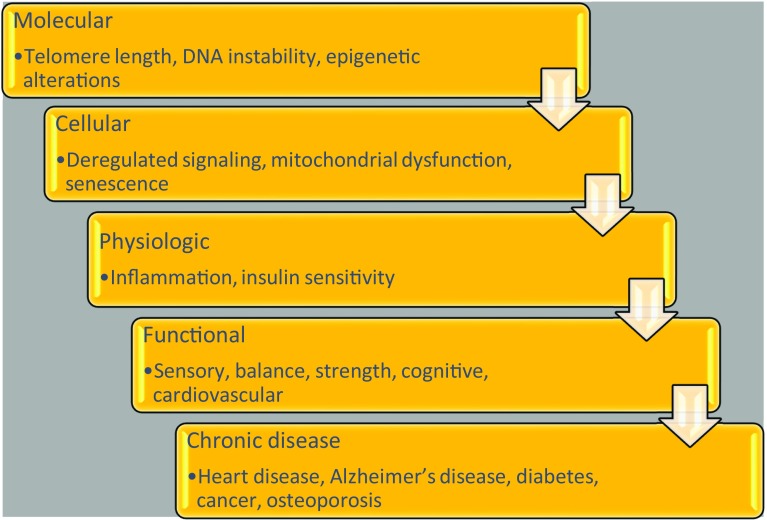
Multifaceted aspects of ageing

Nevertheless, understanding the phenomenon of ageing is critical to ameliorating its impact on both individuals and society. Ageing has been described as “…a decline or loss (a “de-tuning”) of adaptation with increasing age, caused by a time-progressive decline of Hamilton’s forces of natural selection…” [131]. However, ageing is considered more like the sum of its parts—a non-linear multifaceted phenomenon, that can be defined at the molecular, cellular, physiologic, functional levels, and by disease state (Fig. 1).

At the molecular level, ageing is perhaps most well defined by telomere attrition, which is believed to determine cellular lifespan. Other molecular factors linked to ageing include genomic instability and epigenetic alterations. These, in turn, can result in declining cellular function in the form of altered intercellular communication, organelle dysfunction and cellular senescence [93]. These changes manifest themselves at the physiologic level resulting in chronic inflammation, or “inflammageing”, alterations in body composition, energy metabolism and neuronal function [162]. Ageing also results in changes in sensory functions, including changes in taste, smell and diminished appetite [160]; and changes in visual and auditory function [78]. Sarcopenia is a rapid age-associated decline in skeletal muscle mass and physical function resulting from a convergence of chronic inflammation, hormonal changes, cellular dysfunction, poor diet and lack of physical activity [161]. Age-related cognitive changes can also serve as precursors to dementia and Alzheimer’s disease [128]. In addition to cognitive disease, ageing also results in an increased risk for a variety of other chronic diseases, including heart disease, diabetes and cancer. According to data from the US Centers for Disease Control, compared to adults 45–64 years of age, those 65 and older have more than twice the prevalence of heart disease, diabetes and cancer [41].

Researchers have recently assessed age-associated changes in the microbiome. The population and diversity of gut microorganisms evolves with age from birth to death. Initially, there is a rapid rise in number and diversity of organisms from infancy to adulthood, followed by a precipitous decline in diversity through the elderly years [83]. The cause for this decline is uncertain and likely multifactorial. Further, it remains to be elucidated what role, if any, the change in microbial diversity has with the cellular, physiologic and functional changes that define the ageing process.

**Fig. 2:**
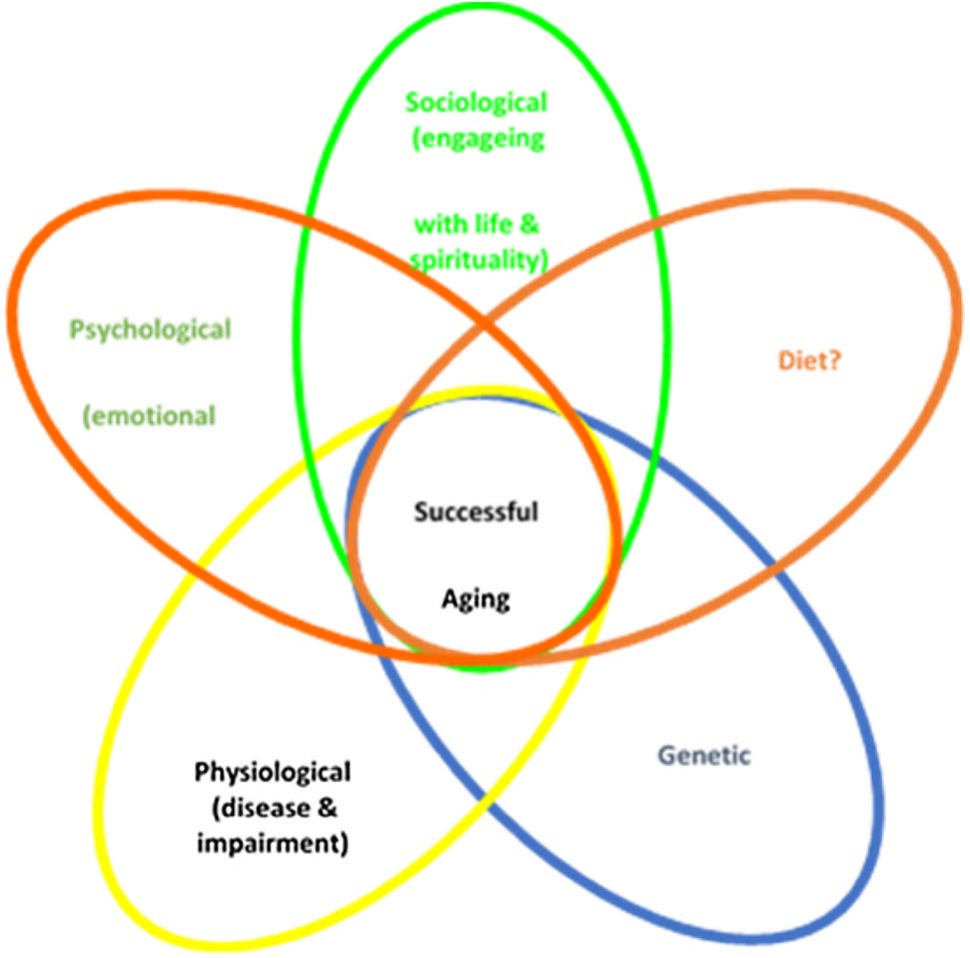
Key factors for healthy ageing

The interest in ageing has progressed from understanding its origins, mechanisms and processes, to studying how to reduce, delay or reverse its effects, and importantly, policy responses that addresses older adults’ needs and rights. This has led to an entire new field of research, new public health initiatives and health and wellness consumer products collectively under the category of “healthy ageing”. Although once thought of as merely the absence of disability and chronic disease with longevity, the term healthy ageing has evolved to mean much more. Today, the term is meant to encompass social well-being and quality of life as well [84]. Despite widespread interest in healthy ageing, there are differing definitions and interpretations of the term, e.g., “successful ageing” or “ageing gracefully”. Irrespective of the term or terms used, healthy ageing has evolved to include the intersection between avoiding and managing disease and disability, optimizing cognitive and physical functions and engagement with life throughout the ageing years [133] (Fig. 2).

Researchers have developed indices that can be used to predict factors, behaviors and traits that best contribute to healthy ageing. For example, Tyrovolas et al. developed a ten-point healthy ageing index, scoring attributes such as education, participation in social activities, number of yearly excursions, adherence to a healthful ‘Mediterranean diet’, frequency of physical activity and BMI. Elderly subjects who scored the highest on the healthy ageing index also reported using less healthcare services [143]. Other strong associations with healthy ageing include non-smoking status, the number of social contacts, better self-perceived health, independent living, life satisfaction and absence of depression [35, 64].

While substantial progress has been made identifying predictors of healthy ageing, assessing the impact of particular interventions has proven more challenging. This is due, in part, to the cost and difficulty of studying the long latency of disability and disease, and the subjective nature of some healthy ageing predictors. Moreover, heterogeneity in the ageing processes reflects peoples’ accumulated opportunities and vulnerabilities, as well as their choices and personal values. Identifying inequalities and identifying social, economic and other policies that can reduce unfair processes within and across countries are key to improving everyone’s chances to optimize healthy ageing. Nonetheless, measuring healthy ageing and the impact of lifestyle interventions remains an area of research focus. Metabolomics and transcriptomics have led to the emergence of biomarkers that may be used to assess the impact of various interventions on the process of healthy ageing [16, 109, 119].

Clearly the role of diet and nutrition is central to the sustaining of life. However, little is known about specific nutritional interventions that most effectively promote healthy ageing. At a recent workshop organized by the National Academy of Science, Engineering and Medicine, only caloric restriction was identified as a well-established dietary intervention that promotes healthy ageing (specifically longevity) [112]. With a rapidly ageing global population and the accompanying increase in comorbidities, mortalities and associated social costs, there is an urgent need to identify solutions including nutritional and dietary interventions that could promote healthy ageing. And despite this broad need, the ability to measure the impact of these interventions remains a major research challenge.

## WHO’s new mandate to measure intrinsic capacities and functional ability across the life course^1^

Society’s response to population ageing requires a vision to harness extra years of life, ensure that these years are spent in good health, and that these can be used to do what people value through-out the life course. A fundamental transformation in policies and institutions is required to enable a cohesive response that celebrates diversity yet narrows health inequities, within and across countries.

The World Health Organization (WHO) published its first World Report on Ageing and Health in September 2015 [154], and all Member States endorsed its first Global Strategy and Action Plan on Ageing and Health (GSAP) in May 2016 [155]. The Strategy represents a commitment from Member States and mandate for the WHO to establish partnerships to implement five strategic areas and reach agreed upon goals, including setting up a *Decade for Healthy Ageing 2021–2030* aligned to Agenda 2030. One strategic area is to “Improve measurement, monitoring and research” with a sub-objective to agree on ways to describe, measure, analyze and monitor healthy ageing and document a baseline across countries by 2020.

The WHO Report and GSAP promote healthy ageing as a person-centered concept, based on life course and capability-based perspectives that can be applied to all people in all settings. Rather than a focus on morbidity or disease, healthy ageing is defined as “the process of developing and maintaining the functional ability that enables well-being in older age, with functional ability determined by the intrinsic capacity of the individual, the environments they inhabit and the interaction between them.” [153].

Functional ability (FA) comprises the health-related attributes that enable people to be and to do what they have reason to value. It is determined by the intrinsic capacity of the individual (i.e., the combination of all the individual’s physical and mental—including psychosocial—capacities). Moreover, ageing is not a disease.

Intrinsic capacity (IC) at any point in time is determined by many factors, including underlying physiological and psychological changes, health-related behaviors and the presence or absence of disease. These in turn are strongly influenced by the environments in which people have lived throughout their lives. Environments comprise all the factors in the extrinsic world (understood in the broadest sense and including physical, social and policy environments) that form the context of an individual’s life. Healthy ageing is inclusive of all older adults, in contrast to “successful ageing” or “anti-ageing” discourses that focus on elimination of disease or ageing processes [107]. Moreover, broader determinants of health and intermediary determinants [135], such as need met by health and long-term care services, are recognized as responsible for a large part of the heterogeneity observed in older age.

**Fig. 3:**
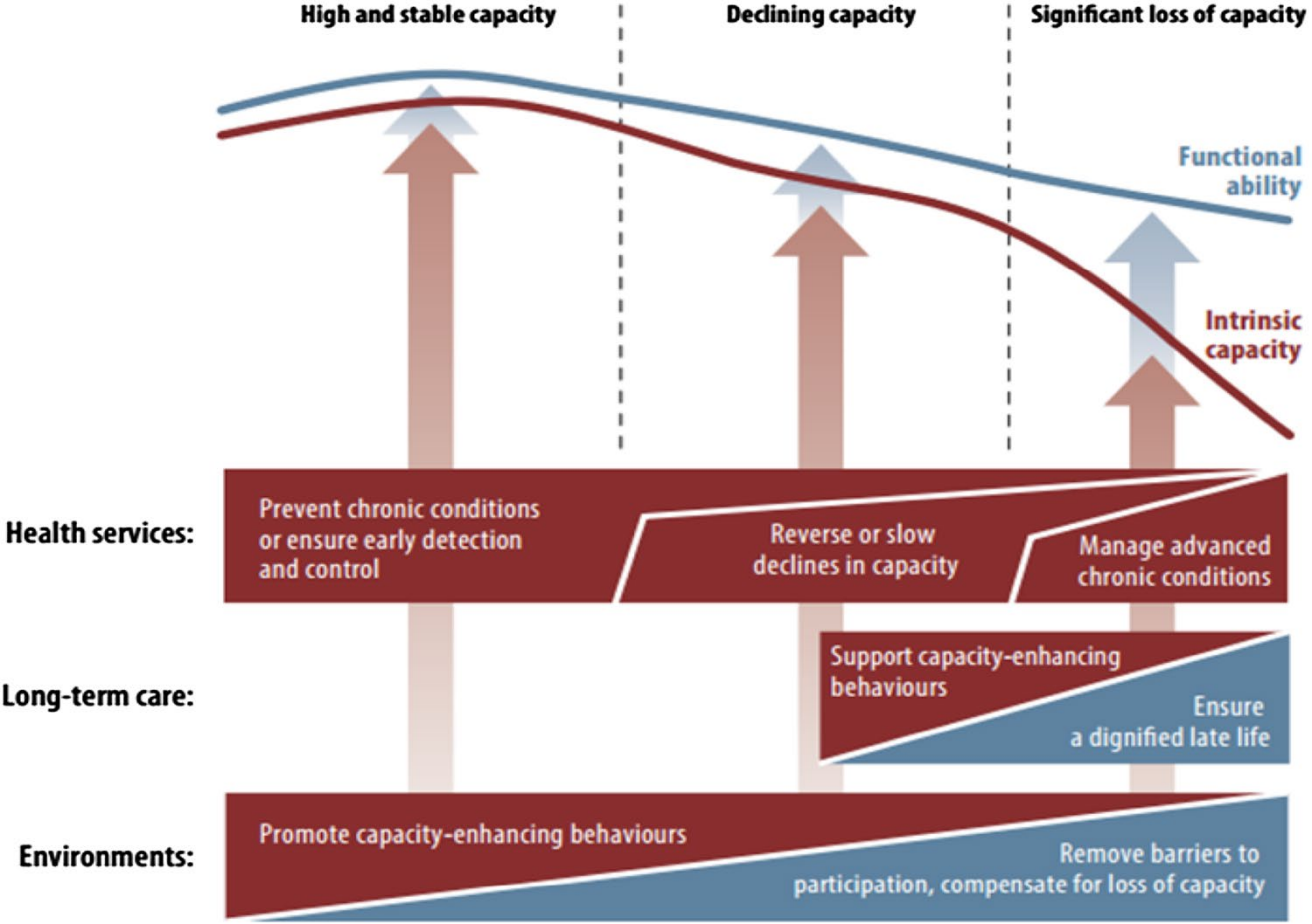
Public health framework for healthy ageing: opportunities for action across the life course

Using these concepts, the World Report proposed a public health framework for action addressing healthy ageing [9]. Figure 3 illustrates conceptually that when considering the population as a whole, IC and FA can vary across the second half of the life course. Trajectories reflect a continuous phenomenon, and can be divided into three common periods: a period of relatively high and stable capacity, a period of declining capacity, and a period of significant loss of capacity. These periods are not defined by chronological age and trajectories are not necessarily monotonic (that is, continually decreasing). Distinguishing between IC and FA is necessary to understand if levels, distributions and trajectories of functioning are due to changes in the individual or the environments they inhabit or both. It is also necessary to document what can be done to improve IC and FA for individuals, groups or populations, involving different policies, sectors and interventions. To agree on ways to describe, measure, analyze and monitor IC and FA, each concept requires further clarification, including a description of what are its components, pathways to optimize each across the life course, measurement approaches, and useful metrics to monitor and communicate progress.

WHO is pursuing a collaborative process to develop and test a standardized approach to measure IC and FA that is person centered. The WHO International Classification of Functioning, Disability and Health (ICF) [152] offers an international reference and normative framework for describing, understanding and studying health and health-related states, outcomes and determinants, that are etiology-neutral and applicable to individuals and populations. This can inform the approach to agree on multi-domain profiles of IC and FA, drawing on the ICF’s universal and standard dimensions of “body functions and body structures”, “activities”, “participation” and “contextual” factors. For each domain, what should be measured needs to meet criteria, such as whether this will convey useful information on a person’s or population’s current IC and FA status, whether this reflects a pre-condition or critical stage to optimize IC or FA in the future, or whether this is sensitive to predict potential declines or improvements. How each of these are actually measured—and their feasibility in clinical or community settings—could be achieved through a range of biomarkers, other measured or performance tests, self-reported questionnaires, or observations. Finally, each domain may be assessed using several measures, then combined for each “domain score” for a multi-domain profile of IC and FA, and then be aggregated across domains, to obtain a composite score for IC and FA, respectively.

Monitoring and eventual evaluation should respond to at least three questions:

- What are the levels and distribution of IC and FA globally and is it getting better?
- What contributes to inequalities, and whether people have their needs and rights addressed?
- What is the impact of policies and actions at different levels (local, national, regional, global) on the average levels and distribution of IC and FA?

As there is no existing single generic instrument for assessing IC and FA, three approaches drawing on existing data mapped to IC and FA, provide preliminary insights to the first question.

Re-analyses of data collected through the WHO Study on Global Ageing and Adult Health (SAGE) [134], Wave 1 (2007–2010), offer an initial attempt to measure IC in nationally representative cross-sectional samples from all six participating countries (China, Ghana, India, Mexico, Russia, South Africa). IC is described through eight domains (mobility, self-care, pain and discomfort, cognition, interpersonal activities, sleep and energy, affect, and vision), and assessed by tests and self-reported questionnaires.

**Fig. 4:**
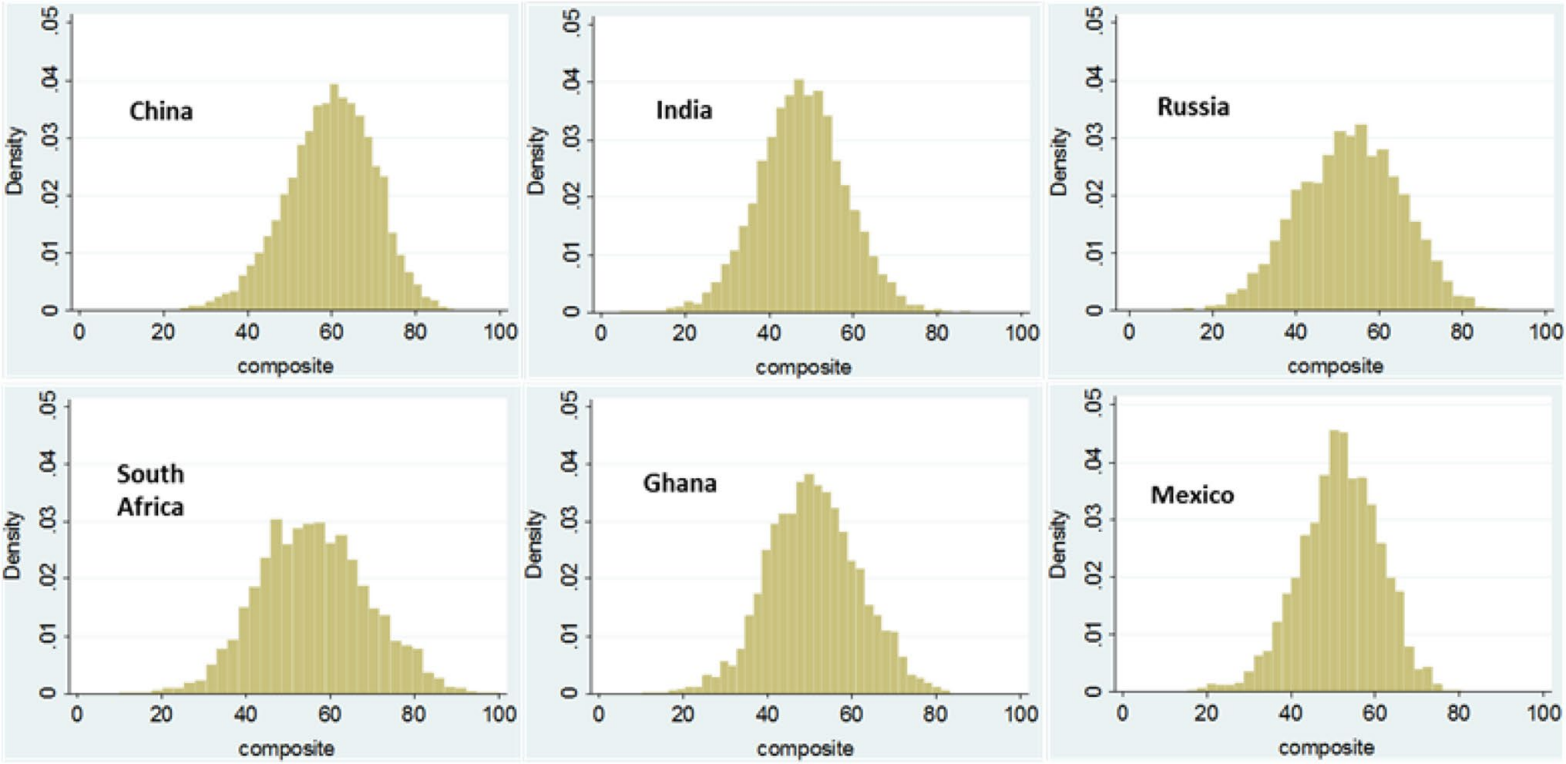
Distribution of intrinsic capacity score for six countries, SAGE wave 1 (2007–2010), ages 50 +, both sexes

For people 50 years and over, Fig. 4 provides population representative distributions (histograms), of the composite IC score from 0 to 100 combining information across each domain, with a higher score representing better IC. Another example is reanalysis of longitudinal data drawn from the English Longitudinal Study on Ageing (ELSA) [32]. One analysis describes IC in five domains (vitality, sensory, locomotor, cognitive, psychosocial), through commonly collected biomarkers and self-reported measures over time; another identifies distinct trajectories of IC over time. Finally, the WHO Model Disability Survey [27], conducted in nationally representative samples in Chile and Sri Lanka, provides the only preliminary approach to quantifying FA in light of hindering or facilitating aspects of the general environment, such as family and social support; attitudes of others, accessibility to information, regular use of medication, personal assistance, assistive products for self-care, mobility, seeing, hearing, work and education, facilitators at home, school, work and community.

Additionally, systematic efforts are needed, including reanalysis of other existing data, such as from the vast network of health and retirement studies, and new research on ways to collect data and develop a generic instrument specifically constructed to monitor IC and FA. To conclude, WHO launched the International Consortium on Healthy Ageing Metrics and Research in March 2017 and it has identified work packages to build up standards on healthy ageing data, measures, and metrics by 2020. Members include academic institutions, other UN and international bodies, non-government organizations, as well as other collaborative networks, e.g., the UN City group on age and age-disaggregated data. Measuring the change, we collectively want to see is part of the fundamental transformation needed across societies to enable healthy ageing.

Given the vast literature, evidence and recommendations on nutrition and older adults, three discussion questions to consider:

1. How can the concepts of IC and FA take stock of the importance and “natural consequences of good nutrition” for healthy ageing—to optimize IC and FA across the life course—particularly the second half of life?
2. Likewise, in the construction of multi-domain profiles of IC and FA—that are etiology-neutral/person-centered, what should be included and how should these be measured? What can be measured in clinical settings, in community settings or where people live?
3. What existing research and monitoring efforts and tools can provide opportunities to re-analyze data and or test new data collection approaches by 2020?

Key references: [151–153].

## Cross-cultural approaches to biomarkers for healthy ageing

Definitions of healthy ageing continue to evolve. The WHO recently conceptualized a public-health framework for healthy ageing that considers physical health, mental health, environment, and environmental support [154]. Priorities for action that can help achieve healthy ageing under this framework include aligning health systems to the older population they now serve, developing systems of long-term care, creating age-friendly environments, and improving measurement, monitoring and understanding. This framework can also be used to support the rapidly growing population of the very old, such as centenarians who are aged 100 and older [140]. This presentation elaborated on WHO’s framework in two ways among centenarians: (1) nutrition as an environmental support to healthy ageing and (2) examination of cross-cultural differences and similarities between centenarians residing in Tokyo, Japan and Georgia, USA, in subjective measures of well-being, physical health, and social health.

### Nutrition in long-lived Georgians (USA)

The Georgia Centenarian Study (USA) has been ongoing since 1988 and seeks to understand how centenarians live longer and to identify specific biological, psychological, sociological, and nutritional characteristics that support an exceptionally long life [126]. Centenarians in Georgia are diverse in terms of race/ethnicity, socio-economic factors, living situation (living in the community vs. nursing homes), and many other factors [125]. Nutrition was examined through food frequency questionnaires and biomarkers of nutritional status in the serum and in post-mortem brain tissue.

Compared to Georgia centenarians in nursing homes, those residing in the community were more than twice as likely to be able to eat without help and to receive most of their nourishment from typical foods [69]. However, those residing in nursing homes had significantly higher exposure to all food groups examined, including dairy, meat, poultry and fish, eggs, green vegetables, orange/yellow vegetables, citrus fruit or juice, and oral liquid supplements. These findings suggest that nursing homes provide environmental support in the form of nourishment for centenarians.

Serum biomarkers of vitamin B12, vitamin D and carotenoid status also were assessed. Adequate vitamin B12 status was significantly positively associated with being African American vs. white, taking dietary supplements with B-vitamins, and not having atrophic gastritis [71], indicating that predictors of vitamin B12 status are related to race/ethnicity, intake from supplements, and preservation of gastric function.

Vitamin D status (serum 25-hydroxyvitamin D) in Georgia centenarians was similar to that for the US population of older adults [70], but much higher than centenarians in Italy where vitamin D fortification of foods is uncommon [121]. It is possible that widespread use of dietary supplements and fortification of the food supply in the US accounts for the similar vitamin D status among Georgia centenarians compared to the US population. In these Georgia centenarians, vitamin D status was higher among whites vs. African Americans, those taking dietary supplements with vitamin D, and those assessed in the summer or fall [70]. Seasonal variation in vitamin D status suggests that very long-lived people may retain some ability to synthesize vitamin D in the skin. Also, higher vitamin D status among Georgia centenarians was significantly associated with higher grip strength, a measure of functional ability [50].

Post-mortem concentrations of brain lutein in the subset of centenarians without dementia were significantly and positively associated with a range of cognitive measures [68]. In contrast, there were few associations of alpha-tocopherol with cognitive function [68].

In summary, several biological and environmental factors are associated with vitamin status throughout life including among centenarians. These factors include race/ethnicity, use of dietary supplements, food fortification, certain foods, and season in the case of vitamin D. Also, measuring, monitoring, understanding, and modifying the nutritional environment are essential for long-lived people.

### Cross-cultural differences in Japan vs. Georgia (USA)

The subset of participants who were cognitively intact were the focus of these cross-cultural comparisons among centenarians in Japan compared to Georgia [111]. There were marked differences in demographic characteristics between the Japanese and Georgia centenarians such as the percent living at home (93 vs. 80%, respectively) and living alone (5 vs. 40%, respectively). Differences in several indices of health were also observed. Compared to the Japanese, the Georgia centenarians had significantly worse cognitive function and better physical function (activities of daily living), but fewer chronic diseases and better vision and hearing.

The Philadelphia Geriatric Center Morale Scale was used to measure subjective well-being. Georgia centenarians reported higher scores on well-being (satisfaction with social relations and psychological comfort). However, these cultural differences in well-being were attenuated after controlling for predictors such as sociodemographic factors and health resources. Regression analyses revealed that health resources (cognitive function, hearing problems, and activities of daily living) were strong predictors of well-being in both countries. Social resources (living with others) were strongly associated with one dimension of well-being (attitude toward one’s ageing) only among the Japanese centenarians. These findings support the existing lifespan and cross-cultural literature, indicating that declines in health impose certain limitations on adaptive capacity in oldest-old age irrespective of cultures, and that social embeddedness is valued in Eastern cultures [111].

These cross-cultural comparisons of centenarians suggest that the environment differs among individuals and among cultures, especially regarding living alone or living in a nursing home, which are both high among the Georgian compared to the Japanese centenarians. Also, findings in the Georgia centenarians suggest that nutrition may be a modifiable environmental factor for physical function (vitamin D) and cognition (lutein). Studies are ongoing to examine associations of cognitive and brain health with additional nutrients in the Georgia centenarians, such as vitamin K, docosahexaenoic acid (DHA), and other fatty acids [68].

## Quantification of biological ageing: implications for clinical trials of interventions to slow ageing and extend healthy lifespan

Interventions to extend healthy lifespan or “healthspan” are needed. Accumulating evidence suggests molecular changes that occur with ageing are among the root causes of age-related disease and disability [80, 93]. Experiments with animals show that these molecular changes can be slowed or reversed, producing increases in healthy lifespan [39, 75]. Translation of these therapies, called “geroprotectors” [110], to extend human healthspan is increasingly plausible [92, 113, 114]. A barrier to translation is the challenge of measuring changes in the rate of human ageing.

Unlike worms, flies, and mice, humans live too long to observe complete lifespans within individual studies. Age-related disease and disability typically develop over the second half of the human life course, a period spanning decades. Interventions that modify biological processes of ageing to prevent age-related disease are, therefore, needed relatively early in life, before age-related disease becomes established [49, 108]. True tests of the effectiveness of such interventions will require decades of follow-up. To establish proof of concept for such long-term studies, measurements are needed that allow tests of a candidate therapy’s potential to slow the rate of human ageing over shorter intervals [12, 73].

Measurements to quantify biological processes of ageing could be implemented to test putative geroprotective effects of interventions over the short term. Measurements taken before, during, and at the conclusion of an intervention could be used to estimate how that intervention might change the rate of age-dependent deterioration in system integrity, providing a simple test of whether the intervention showed promise to extend healthspan. Measurements to quantify biological processes of ageing are now being developed. The most promising combine multiple sources of information, e.g., from clinical parameters or gene expression and DNA methylation measurements [74]. Initial epidemiologic studies of these algorithm-based biomarkers of ageing indicate promise [74]. For example, so-called “epigenetic clocks” composed of dozens or hundreds of methylation marks have been shown to predict mortality in multiple studies [24]. Research is needed to test if these new ageing biomarkers can inform evaluations of candidate therapies to slow ageing and extend healthspan [13].

Work by Belsky et al. [10–13] has focused primarily on measures of biological ageing derived from indices of organ system functioning, including blood chemistries, blood counts, and organ system tests such as blood pressure, lung function, and cardiorespiratory fitness measurements. This work has yielded four main findings. First, consistent with theories of biological ageing, organ systems throughout the body show age-dependent declines in integrity even among young healthy people in their 20s and 30s. Moreover, the rate of this decline is correlated across different organ systems and is variable between individuals. Thus, measurement of the rate of biological ageing in relatively young people as the average rate of decline in integrity across organ systems is possible [11]. Second, young people whose bodies exhibit a faster rate of biological ageing measured in this way have worse physical functioning, as measured by tests of strength, balance, and motor coordination, and show evidence of early cognitive decline, as measured from changes in cognitive test performance between childhood and midlife. They also report being in worse health and are rated as looking older by others [11]. Third, people with early-life characteristics associated with shorter healthy lifespan, including exposure to childhood poverty and child maltreatment, poor health in childhood, and low childhood cognitive function and deficits in self-control evidence a faster rate of biological ageing [10]. Fourth, the rate of biological ageing measured from decline in the integrity of multiple organ systems is slowed by caloric restriction, an intervention established to extend healthy lifespan in animals [12]. This last study observed changes in the rate of biological ageing over the relatively short term of a 2-year intervention trial.

Critically, Belsky et al. [10–13] observed consistent findings for multiple approaches to quantify biological ageing from organ system function data using their own longitudinal-change “Pace of Ageing” approach and cross-sectional methods that compare research participants to reference populations to quantify their biological age [82, 90] or their homeostatic dysregulation [29]. These methods, which can be implemented using standard blood chemistry panels and other data routinely collected in clinical studies, suggest new possibilities for studies to evaluate interventions that may affect the rate of ageing. In theory, these measures of biological ageing may be more sensitive than individual disease-endpoint measures, which focus on more extreme outcomes. Instead, biological ageing measures are designed to capture subtle, organism-wide shifts in physiological integrity. They may thus provide an interesting avenue for studies of nutritional interventions.

## Public health nutrition in the data age: opportunities, pitfalls, and future perspectives

To study healthy ageing, data-driven tools and models can be used to quantify nutrition and health in a population. The value of good data can be stressed as it plays a key role in today’s data age, including the use of secondary data sources. To assess intakes at a population and subpopulation level, the data collected need to be of high quality, while keeping in mind time, cost, participant burden and other factors. The opportunity to analyze specific demographics, including vulnerable groups from a nutritional standpoint, is of great importance when trying to address healthy ageing. Food consumption surveys provide extensive information. They enable the assessment and monitoring of health and nutritional status of specific demographics, to inform healthy eating guidelines and to reduce diet-related chronic diseases, which all have a part to play in healthy ageing.

Food consumption surveys are also used to evaluate the benefits and safety of potential supplementation and fortification strategies, as well as to inform businesses on consumer intakes, dietary impact of products and ingredients, as well as research and development decisions. In addition, the data are used for monitoring food safety via food exposure assessments to additives, pesticides and contaminants. When analyzing and modelling those specific topics, it is of importance to have access to individual food diaries, detailed and quality food composition or chemical data, demographic, anthropometric and biomarker data. Methodologies for assessment of food and supplement intake vary widely, however, there is a view to harmonizing them within the European Union (EU) [2].

Even though the acceleration in data generation presents opportunities, gaps in availability or access, unfit-for-purpose data, lack of specific information and out-of-date data remain an outstanding challenge for public health nutrition research. To overcome such problems, data sources including market research data, online tools and platforms to gather data more efficiently, as well as the construction of new models combining complementary data from various sources of origin can be promising alternatives.

Models such as the Compiled European Food Consumption Database [116] and the Global Expanded Nutrient Supply (GENuS) Model [138] extrapolate consumption using existing databases. The Compiled European Food Consumption Database estimates the consumption of the European population using European Food Safety Authority (EFSA) comprehensive summarized intake statistics [98] and simulating 29 days of intake distributions for 40,000 individuals using 36 clusters of age groups and gender having similar diets. A limitation is that the database contains no estimates of nutrient intakes and no breakdown by country. Also due to the applied methodology, some outliers may be over- or underestimated. The GENuS model uses Food and Agricultural Organization (FAO) food balance sheets, production and trade data combined with nutrient composition tables to calculate the nutrient supply across 175 nations, 26 demographic groups and 225 food categories. The database, however, does not assess consumption of foods at the individual level and nutrient availability is overestimated. Examples of ongoing efforts to gather up-to-date and more harmonized data on food consumption are the EU menu [2] and the International Dietary Data Expansion (INDDEX) project [28], but whether the databases will be accessible and how they can be used remain to be seen.

The use of large datasets and mathematical models to assess and define optimal dietary changes in specific populations can generate valuable insights and opportunities for public health nutrition strategies and food product development.

Consumption of bioactive compounds can have beneficial and/or adverse health effects; however, intakes of those compounds are not routinely assessed in populations. As part of the European Commission (EC)-funded BACCHUS, ‘Beneficial effects of bioactive compounds in humans’ project, national food consumption data were linked to bioactive composition data. To estimate intake distributions and to account for variability of bioactive concentrations, a probabilistic intake modelling approach was applied [5, 124].

In the EC-funded ODIN project on vitamin D entitled Food-based solutions for optimal vitamin D nutrition and health through the life cycle; EC Contract 613977 [117], national food consumption surveys and a standardized vitamin D database were combined to assess vitamin D nutrition. Incremental food fortification scenarios, such as enrichment of animal food sources (meats, eggs, fish and dairy products) were then applied to ensure the safe increase of vitamin D intakes across the population distribution and prevent deficiency. In addition, safety across European countries was assessed using available data on consumption [98] and a worst-case scenario approach due to the lack of individual data [123].

Another example examined new food products and their impact on health outcomes, such as blood pressure and cardiovascular events, by assessing individual data on food consumption combined with intake models. This study by Dainelli et al. [31] looked at the shift in intakes of potassium-fortified milk powder and consecutively the health impact via replacing milk consumption with the fortified product in adults above the age of 45.

As part of the overall Healthy Ireland initiative [53], the Irish food industry is proactively contributing to healthier choices and product reformulation, but progress is not assessed regularly when looking at consumption. To quantify the impact of voluntary food reformulation efforts on Irish consumers, probabilistic intake assessments were performed using Irish national food consumption surveys (IUNA) in combination with industry data on reformulated product composition and market share data [60–63, 122].

Combining databases and probabilistic intake models represents a great opportunity for informing public health strategies, including healthy ageing. The impact of dietary changes via using and modelling databases can provide powerful information for government and industry; however, available and fit-for-purpose databases, as well as the tools and expertise to exploit such data remain a challenge.

## Public health implications of immunosenescence: role of nutrition

Since January 1, 2011, every day for the next 20 years, roughly 10,000 Americans will celebrate their 65th birthday. The US Census Bureau projects that between 2005 and 2025, the number of US individuals > 65 will increase by 50% [150]. Globally, currently 686 million people (12%) are over 60. By 2050, it is predicted that there will be nearly as many people aged > 60 as children under 15. In many countries, life expectancy of age 60 is now at least a third more than what it was in the mid-twentieth century [55]. Interestingly, the over-80 group is projected to be the fastest growing subset in this over-65 trend, which was 14% in 2012, and is predicted to be at 20% in 2050 [150]. This represents a major demographic shift with significant public health and socio-economic impact. Advances in science have greatly increased lifespan; however, at the same time, new and developing challenges, such as sarcopenia, cardiovascular disease, obesity, diabetes, dementia, macular degeneration, cataracts, and emerging infections, as well as the costs associated with these conditions are on the rise, impacting the health and functional lifespan of older adults while diminishing their ability to be fully contributing members of their communities.

Accumulating evidence, however, indicates that poor health in late life is not inevitable. Contrary to the previously held belief that increased risk of diseases and disability with advancing age results from inevitable, as well as genetically determined intrinsic ageing processes, more recent studies indicate that many of the usual ageing characteristics are due to lifestyle and other modifiable factors and are not unavoidable consequences of ageing itself [42, 51, 66]. Thus, developing strategies to increase “health span” or the years of “successfully ageing” for older adults becomes critical—socially, and economically.

**Fig. 5:**
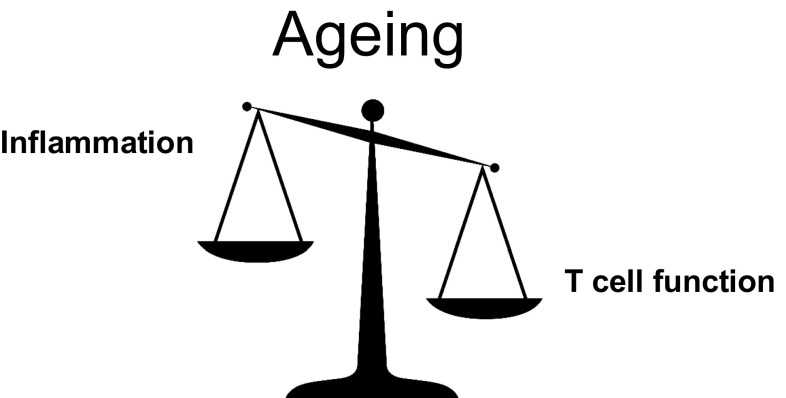
Ageing is associated with dysregulated immune and inflammatory responses exhibiting increased inflammation on one hand and decreased T-cell-mediated function on the other hand

Studies across species show that ageing is associated with dysregulated immune and inflammatory responses, which may contribute to many age-related diseases including cancer, infection, cardiovascular diseases, diabetes, Alzheimer’s disease and osteoporosis. The immune system is comprised of different cell types that engage in a complex series of interactions to defend the host against invading pathogens. These interactions, under normal conditions, are well-orchestrated so that a temporary upregulation in inflammatory responses needed to eliminate the pathogen is subsequently diminished and controlled. With ageing, the normal “checks and balances” of the immune response is impaired, creating a state of chronic inflammation (hyperactivity of parts of the immune system involved in innate immune response) on one hand, and hypoactivity of the cell-mediated immunity, particularly T cells, on the other (Fig. 5).

This dichotomy presents a challenge in devising effective interventions to prevent/treat age-related changes of the immune response. Recent evidence, however, suggests that both the hyper- and hypo-activity of immune response associated with ageing might be governed by some of the same molecular/biochemical aberrations that might be responsive to nutritional intervention.

A significant portion of the global older population have nutritional problems exhibited as both under-nutrition (e.g., micronutrient deficiencies such as B vitamins, vitamin C, E, D, Se, Zn, Ca, and Fe) and over-nutrition (i.e., obesity); often existing together [81]. These age-associated nutritional problems provide opportunities and challenges in developing interventions that could reduce inflammation while improving host defense against infection. Strategies include single nutrient interventions, e.g., vitamin E [20, 46, 48, 52, 56, 99, 103, 105], vitamin B6 [106], zinc [6–8, 43, 100, 126], fish oils [102, 104, 159], or whole food such as wolfberry [38, 127, 145, 146] and pre-and probiotics [40, 87]. In addition, calorie restriction in both animal and humans has been shown to reduce inflammation and improve immune response [3, 101, 115, 149]. These studies provide strong evidence that appropriate nutritional intervention could optimize the immune and inflammatory responses in older adults leading to improved resistance to chronic and infectious diseases. These interventions, particularly started earlier in life, could have significant public health implications for older adults. However, success has been curbed due to the lack of adequate information on specific nutritional needs of older people. As a result, older people of a wide age range from 60 to 100 and with varied genetic and socio-economic backgrounds are included in studies without appreciation for heterogeneity of their nutritional status, which in turn, reduces the effectiveness of any given intervention. Addressing this gap, would expedite development of efficient and cost-effective nutritional strategies to improve quality of life in older adults.

## A public health perspective on nutrition and its impact on quality of life

Despite the obvious connection existing among ageing, nutrition and quality of life (QoL) in older adults, a systematic investigation in the field is lacking [37]. This is particularly the case when the focus is moved from clinical-based studies, where QoL is a traditional outcome of clinical or nutritional intervention, to the broader context of public health. Public health implications of nutrition and QoL in the older stages of life are of paramount importance, being interconnected to all of the most prominent issues, from (1) multi-comorbidities enhanced by poor diet and nutrient deficiencies, (2) polypharmacy as a consequence of the former situation and as a sign of improper approaches to healthy lifestyles, to (3) social aspects such as meal sharing with friends or families and (4) economic aspects, such as food insecurity.

**Fig. 6:**
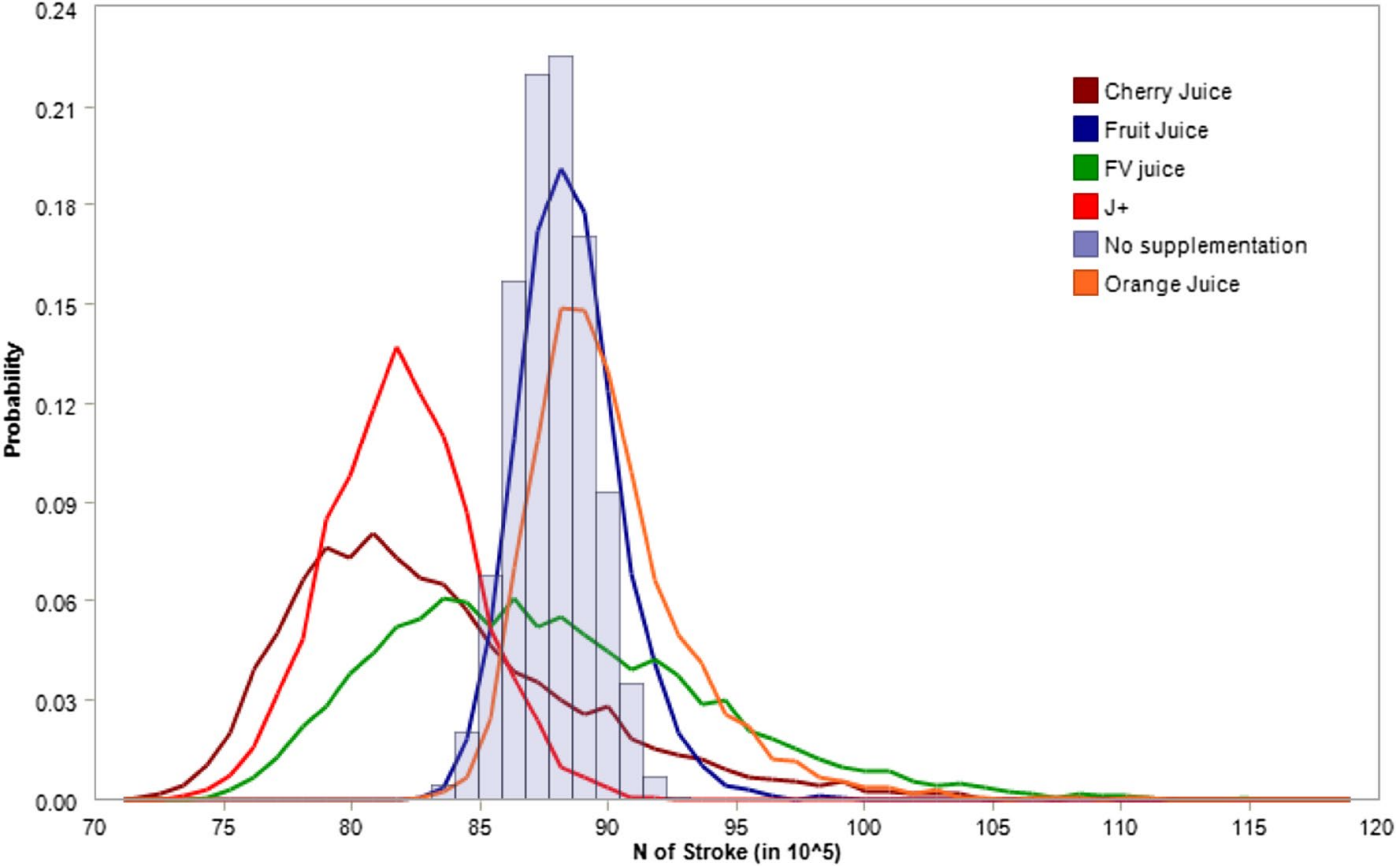
Distribution of expected number of stroke events without (gray bars) and with concentrates supplementation (smoothed curves). Data are Monte Carlo microsimulations referred to the decade 2015–2025 for US

Not only has better diet in older adults been shown to be associated with significantly higher physical and emotional QoL scores [147] and with better functional status [45], but the whole life satisfaction in older adults is impacted by nutritional adequacy [79]. The definition of healthy ageing itself includes diet quality and eating habits as essential components [121], with immediate consequences on hard outcomes, such as overall and disease-specific mortality [89]. Diet quality, which is mainly defined in terms of proper nutrient variety and adequate caloric intake, is, however, rarely met in older adults, mostly because of the aforementioned issues. In particular, intake of fruits and vegetables, which provide a wide variety of different micronutrients and bioactive compounds, are sub-optimally consumed by older people, with no improvement over the past decade. Supplementation has been advocated as a valid remedy to ameliorate diet quality challenges in older adults, most often in a very cost-effective way. In a recent review (unpublished)^2^ on the impact of reported use of the active administration of any dietary supplement (vitamins, minerals, herb or botanical compounds, amino acids, dietary substances, concentrates, metabolites, constituents or extracts), the analysis of 83,350 subjects showed reported supplement use was associated with a significant reduction in mortality risk for cancer (pooled estimated HR 0.93, 95% CI 0.88–0.99) and stroke (pooled estimated HR 0.97, 95% CI 0.95–0.99). A marked impact on stroke mortality was noted for *Ginkgo biloba*-containing supplements (HR 0.59, 95% CI 0.37–0.93), Selenium + Coenzyme Q10-containing supplements (HR 0.46, 95% CI 0.33–0.64) and vitamin B (HR 0.75, 95% CI 0.62–0.91). Alternatively to a single-component analysis of the effects of supplementation on health, a recent review on fruit and vegetables concentrates and their potential impact on risk reduction for major health events [47] showed a strong inverse relationship between reported consumption and major chronic disease, in particular coronary heart disease and stroke (unpublished)^3^ (Fig. 6). Thus, the potential gain in terms of events avoided due to a better nutrition, via supplementation, in older adults, suggests a potential reduction of public health expenditure.

The same simulation scenario as in the previous case shows indeed that a significant number of cases were avoided (approximately 9,965,819 (95% CI 643,567–25,202,911)), attributable to nutrient supplementation (in people aged 65 years or more) (Table 1). In this scenario, fostering vitamin consumption via fruits and vegetables or by supplementation is an essential component for promoting healthy ageing. Supplementation could thus be seen as a cost-effective solution to overcome behavioral obstacles typical of older age yet ensuring proper intake of most relevant nutrients.

**Table 1:** Simulated savings in US due to the usage of supplements

	Age–class	No supplementation	Beta-carotene	C	E	B9	ACE
Overall Health Care Expenditure (US$)	65–84	**403.294** (174.347; 887.958)	402.078 (174.267; 871.077)	360.849 (168.383; 785.064)	399.338 (173.627; 851.128)	391.886 (171.277; 844.23)	392.184 (172.092; 821.755)
	85 +	92.353 (40.706; 203.283)	91.796 (40.695; 199.223)	85.913 (40.187; 167.567)	90.63 (40.564; 202.015)	90.522 (40.667; 193.803)	89.342 (40.482; 192.82)

Numbers are billions US$. Computation referred to US population 2016 and extrapolated to the decennium 2015–2025 on the estimates based on Medical Expenditure Panel Survey [54]

## Life in the margins: the health impact of micronutrient insufficiency

Micronutrient deficiencies are common in older adults around the globe [157]. Numerous factors can limit the ability of elders to access and consume nutrient-dense foods, such as declining income, impaired mobility, poor oral health, altered taste and smell, loss of appetite, lack of food variety, changes in cognition and diminished vision. The greatest burden of chronic disease is borne by older adults, which by their very nature can lead to a disruption of the immune system, changes in metabolism, and impact inflammatory mediators, which can increase the body’s need for specific micronutrients. Medications used to treat chronic disease can be nutrient wasting, causing significant declines in the status of micronutrients vital for well-being and health.

While frank nutrient deficiency states are well-known (e.g., rickets, scurvy, pellagra), there is a growing body of evidence showing that less than optimal biochemical levels are associated with impaired cognitive function, cardiovascular disease, cancer, poor bone health, eye disease and other conditions that are common in older populations [22]. The focus of this presentation was to examine the prevalence, impact and risk factors for several key micronutrients in the ageing population.

### Vitamin B12

The risk for vitamin B12 deficiency increases with age. Using data from the National Health and Nutrition Examination Survey (NHANES), 6.9% of US adults aged 51–70 years and 15% of those over 70 years are B12 deficient [17]. Similar findings were reported in Germany, with 27.3% of people aged 65–93 having deficient serum B12 levels [30]. The decline in gastric acid secretion that occurs with advancing age can make it difficult to absorb food-bound B12 in meat, poultry, seafood, dairy, and eggs. For this reason, the Institute of Medicine recommends adults over the age of 50 get their B12 from fortified foods and/or supplements [59].

The risk for low B12 in elders may be further compounded by the widespread use of proton pump inhibitors (PPI) and histamine H2 blockers, which dramatically inhibit gastric acid secretion and the absorption of B12 [96]. A 2015 meta-analysis found an 80% increased risk of deficiency after 10 months of regular PPI use [72]. Many older adults are prescribed a PPI to prevent gastric bleeding secondary to the use of aspirin, anticoagulants, and/or non-steroidal anti-inflammatories. These drugs are used in the management of cardiovascular disease and pain, two conditions that disproportionately affect older adults.

The risk for diabetes also increases with age. Of the 30 million Americans with type-2 diabetes, 12 million are over the age of 65 [76]. Metformin, a medication commonly prescribed for the treatment of type-2 diabetes, reduces serum B12 levels and worsens diabetes-associated neuropathy [120]. Many patients taking metformin are not monitored or screened for B12 status [23]. It is not uncommon in clinical practice to see a 70-year old patient on both long-term metformin and PPI.

B12 deficiency can lead to difficulty walking, tingling/numbness in hands and feet, fatigue, shortness of breath, loss of appetite, joint pain, depression, loss of taste and smell, cognitive impairment, and dementia [118]. Both patients and clinicians may assume that some of these signs and symptoms are simply the result of ageing. Screening for B12, including methylmalonic acid (a more sensitive indicator of B12 status) should be considered in any patient experiencing these symptoms, who are vegetarian or vegan, or with long-term metformin and/or PPI use [85, 96, 120].

### Vitamin D

Optimal vitamin D status is necessary for the absorption of calcium and phosphate required to preserve mineral homeostasis and bone health, as well as maintaining skeletal muscle performance [26]. While hypovitaminosis D is common worldwide, it is more common and severe in elders [21] due to both environmental and biological factors. Impaired mobility and residential care often limit time spent outdoors and decreased synthesis of vitamin D in the skin makes it difficult to maintain adequate levels even with sun exposure. As ageing advances, intestinal resistance to 1,25(OH)2D impairs the uptake of calcium and a decline in renal function reduces vitamin D activation [33]. Thus, the Institute of Medicine recommends that adults over age 70 take ≥ 800 IU per day (20 µg) of vitamin D3 and increase calcium from 1000 mg per day to 1200 mg per day for women over age 50 years and men over 70 years [58].

Osteoporosis is a silent disease and a major health concern given the ageing of the global population. It is responsible for more than 8.9 million fractures annually worldwide [67]. Falls are a major cause of fractures and are more common in older adults. Roughly 30 % of people over age 65 fall annually, with 10–15% of these falls resulting in fracture [77]. Sixty percent of those who fell in the previous year, will fall again [141]. Seventy-five percent of hip, spine and distal forearm fractures occur among those 65 years or older [97]. Fragility fractures are associated with decreased quality of life, increased disability, more frequent hospital admission and an increased risk of mortality [88]. While a multimodal approach is important for fall protection, vitamin D supplementation alone, or in combination with calcium, has been shown to significantly reduce the risk of falling in elders [142].

A meta-analysis that included eight studies with 30,970 participants conducted by the National Osteoporosis Foundation found that calcium plus vitamin D supplementation resulted in a statistically significant 15% reduced risk of total fractures and 30% reduced risk of hip fractures [148]. This translates into large economic savings, decreased morbidity and mortality, and improved quality of lives.

Given the data showing the beneficial effects of vitamin D on falls and fracture, it is somewhat surprising that many patients who experience a fragility fracture are not recommended vitamin D. In one Italian study, most patients over age 65 years who had a hip fracture (98.2 and 88.3% in 2011 and 2015, respectively) did not receive vitamin D supplementation at the time of the fracture and only 30–35% were receiving vitamin D supplements 1 year after the fracture [26]. This is unfortunate as supplementation may reduce the number of subsequent fractures, enhance muscular strength, and improve balance [25].

Numerous medications can lead to low bone mineral density and an increased risk for fracture. Statins may cause a decline in vitamin D levels and lead to smaller increases in serum 25(OH)D concentration even with supplementation [18, 19]. Anticonvulsant medications accelerate vitamin D metabolism and increase the risk for fracture [65]. Glucocorticoids, aromatase inhibitors and androgen deprivation treatments all significantly increase the risk for osteoporosis and fracture. The American Geriatrics Society recommends against the use of PPIs for longer than 8 weeks in older adults, except in high-risk patients, due to the potential risk of bone loss, fractures and risk of *Clostridium difficile* infection [4].

Clinicians should assess 25(OH)D levels in those with risk factors for low vitamin D, correct any deficiency, and maintain serum levels ≥ 30 ng/mL (75 nmol/L), as recommended by the Endocrine Society [57]. Vitamin D supplementation beyond what is currently recommended is often necessary to correct an underlying deficiency. While the safe upper limit for vitamin D supplementation has been set at 4000 IU per day for most adults [58], clinicians generally use 50,000 IU per week or 6000 IU per day for 8 weeks, or longer, to correct deficiency states [57].

Dietary calcium intake should be assessed with a goal of 1000–1200 mg per day. Patients with chronic kidney disease should be closely monitored for vitamin D and calcium intake to avoid elevated serum calcium.

### Magnesium

Low magnesium intakes and low serum magnesium levels are associated with many of the chronic conditions that plague older adults: type 2 diabetes, metabolic syndrome, chronic inflammation, high blood pressure, atherosclerotic vascular disease, sudden cardiac death, osteoporosis, and colon cancer [129]. Almost half (48%) of the US population consumed less than the recommended amount of dietary magnesium in 2005–2006; down from 56% in 2001–2002 [130]. The Canadian Health Measures Survey (2012–2013), found that 9.5–16.6% of Canadian adults had a serum magnesium below the lower reference cut-off [14].

Evidence from epidemiological studies, randomized controlled trials, and meta-analyses suggest an inverse association between magnesium and cardiovascular disease [132]. A 2013 meta-analysis that included 16 studies with more than 313,000 participants found higher blood levels of magnesium (per 0.2 mmol/L increment) were associated with a 30% lower risk of cardiovascular disease [34]. Magnesium supplementation reduces plasma C-reactive protein (CRP) concentrations in those with levels > 3 mg/dL, which is indicative of inflammation and an increased risk for cardiovascular disease [137].

Magnesium is important for maintaining healthy blood pressure and supplementation (365–450 mg/day) has been shown to significantly lower blood pressure in those with insulin resistance, prediabetes, and other chronic diseases [36]. This is important given that some anti-hypertensive medications (e.g., thiazide diuretics) can cause hypomagnesemia, decreasing their effectiveness and increasing the risk for heart arrhythmias [91].

As mentioned previously, diabetes is a major public health concern, with the global prevalence increasing from 4.7% in 1980 to 8.5% in 2014, and continuing to rise [156]. Insulin resistance has been shown repeatedly to decrease magnesium levels and diabetics with low magnesium show a more rapid disease progression and an increased risk for diabetes-related complications. A vicious forward feeding cycle is created. Magnesium supplementation has been shown to improve glucose metabolism and insulin sensitivity in those with type-2 diabetes [44].

Certain medications can cause a decline in magnesium. The Food and Drug Administration has mandated a warning that long-term use PPI can cause dangerously low magnesium levels and thiazide diuretics, often a first line treatment for hypertension, can cause a decline in magnesium, as well as potassium.

While serum magnesium is a useful screening tool, red blood cell magnesium testing is considered more accurate and should be considered in patients with risk factors for hypomagnesemia. Magnesium supplementation (200–500 mg per day) is generally considered safe; however, clinicians should be cautious in those with diminished renal function.

Given the ageing of the global population, it is imperative that health policy advocates, governmental officials, clinicians and the public be made aware of the importance of micronutrients in overall health, particularly in older adults. Clinicians must have more training in how to identify potential nutrient deficiencies and what testing is most appropriate for determining the status of specific nutrients. We urgently need more research to determine the “optimal” reference range for key micronutrients in older adults, as well as making nutrient testing more widely available, more economical, and reimbursed by insurance and government programs. Otherwise, we will continue to see many older adults living in the margins, when it comes to their micronutrient status.

While the consumption of nutrient-dense food is the foundation for obtaining nutrients, it is simply not enough in certain cases, such as vitamin D. Given the key role that calcium and vitamin D play in maintaining bone and skeletal muscle health, nutrient status should be more rigorously evaluated by the clinician and supplementation more widely recommended. With a fragility-related fracture occurring every 3 s, this inexpensive approach should become part of standard practice in primary and geriatric care for the management of osteoporosis [67].

The widespread use of prescription drugs for the management of many chronic health conditions can also make it difficult to maintain adequate levels of specific nutrients. PPI drugs are one of the most commonly prescribed medications and are also available over-the-counter in the United States. The long-term use of these drugs can increase the risk of fracture, cause magnesium levels to plummet, and interfere with B12 absorption, as well as increasing the risk of *C. difficile* infection. With the increasing prevalence of type-2 diabetes, we will continue to see an increase in prescriptions for metformin, a drug known to deplete vitamin B12. While these medications can be incredibly beneficial, many older adults are taking these drugs long term without any monitoring of micronutrient status. Many clinicians are unaware of the potential for nutrient wasting associated with medication use and simply are not looking for it.

Given that even marginal micronutrient status can adversely affect muscle, joint, and eye health, as well as the immune, cardiovascular and neurological systems, there is an urgent need for better evidence-based guidelines, education and communication with public health officials, medical professionals and the public.

## Conclusion

The annual CRN-International Scientific Symposium and subsequent conference report is continuing to explore and refine scientific content related to optimal nutrition and a healthy lifespan. It is apparent that they are related, most likely with the former affecting maximal length and achieving a lessening of the “…decline or loss of adaptation with increasing age…” Although the nine subject-matter experts provided their insights and in some cases, laboratory-derived data points applicable to healthy ageing, it is also evident that the surface has been barely scratched and that many additional definitions, end-points, markers of ageing can be proposed, and that there are likely many well-conceived possible lifestyle options that can be modified to achieve improved health and optimize the lifespan. One theme that resonates through this entire report is that ageing is NOT a disease. The phrase “successful ageing” does not do justice to the inclusive opportunity that drive an individual to achieve their optimal lifespan. Quality is equally important to the quantity of life years deemed appropriate for each individual, given both controllable and uncontrollable impacting parameters. Most of the presenters either called out terms such as functional ability and intrinsic capacity, or in their oral and written contributions drew tangential points to the health-related attributes that enable a lifespan value option affected by the underlying physiological and psychosocial factors, health and lifestyle-related behaviors and the presence/absence of disease and rapidly-developing decrements. Finally, the mental picture of a life course can be intuitively described with adjustments in internal (genetics) and external (environment) elements over the lifespan affecting the slope and trajectory of a life, a progress towards death that is overlaid by debilitating life stages associated with perceptions of unhealthy and unforgiving “old age”.

## Footnotes

1 R. Sadana is a staff member of the World Health Organization and contributed section on WHO’s new mandate to measure intrinsic capacities and functional ability across the life course. All listed authors alone are responsible for the views expressed in this publication and they do not necessarily represent the decisions, policy, or views of the World Health Organization.

2 Papers screened on PubMed & EmBase for trials with sample size greater than 200 subjects (aged at least 50 years old, defined as healthy or without specific diagnosis, or with most common ageing diseases—cardiovascular diseases, age-related macular degeneration, dementia or diabetes). Outcomes considered were at least one of the following outcomes: all causes mortality, CV death, major CV events (composite endpoint, stroke, myocardial infarction, revascularization, etc.), and cancer.

3 The review focused on seven “concentrates” (juice plus—J+, orange juice fruit and vegetable juice—FV juice, fruit juice, cherry juice, Jerusalem artichoke juice, Artichoke leaf juice). These compounds were analyzed in terms of their impact on measurable physiological outcomes (total cholesterol, homocysteine, systolic blood pressure, body mass index and tumor necrosis factor *alpha*), widely accepted in literature as risk factors for cardiovascular NCDs. Literature review results provided input for Markov chain simulation model, calibrated on US 2015–2025 general population, population with metabolic syndrome and hypertension. Effect of concentrates was thus simulated by linking the direct effect of each concentrate on a clinical surrogate with the effect of the same surrogate on stroke.

## References

[1] Aggett PJ, Hathcock J, Jukes D, Richardson DP, Calder PC, Bischoff-Ferrari H, Nicklas T, Mühlebach S, Kwon O, Lewis J, Lugard MJF, Prock P (2012). Nutrition issues at Codex: health claims, nutrient reference values and WTO agreements: a conference report. Eur J Nutr.

[2] Aguilera J, Gomes AR, Olaru I, European Food Safety Authority (2013). Guidance on the EU Menu methodology; Principles for the risk assessment of genetically modified microorganisms and their food products in the European Union. Int J Food Microbiol.

[3] Ahmed T, Das SK, Golden JK, Saltzman E, Roberts SB, Meydani SN (2009). Calorie restriction enhances T-cell-mediated immune response in adult overweight men and women. J Gerontol A Biol Sci Med Sci.

[4] American Geriatrics Society 2015 Beers Criteria Update Expert Panel (2015). Updated beers criteria for potentially inappropriate medication use in older adults. J Am Geriatr Soc.

[5] BACCHUS. http://www.bacchus-fp7.eu/. Accessed on 3 Jan 2018

[6] Bao B, Prasad AS, Beck FW, Snell D, Suneja A, Sarkar FH (2008). Zinc supplementation decreases oxidative stress, incidence of infection, and generation of inflammatory cytokines in sickle cell disease patients. Transl Res.

[7] Barnett JB, Dao MC, Hamer DH, Kandel R, Brandeis G, Wu D (2016). Effect of zinc supplementation on serum zinc concentration and T cell proliferation in nursing home elderly: a randomized, double-blind, placebo-controlled trial. Am J Clin Nutr.

[8] Barnett JB, Hamer DH, Meydani SN (2010). Low zinc status: a new risk factor for pneumonia in the elderly?. Nutr Rev.

[9] Beard J, Officer A, Araujo de Carvalho I, Sadana R, Pot AM (2016). The world report on ageing and health: a policy framework for healthy ageing. Lancet.

[10] Belsky DW, Caspi A, Cohen HJ, Kraus WE, Ramrakha S, Poulton R, Moffitt TE (2017). Impact of early personal-history characteristics on the Pace of Aging: implications for clinical trials of therapies to slow aging and extend healthspan. Aging Cell.

[11] Belsky DW, Caspi A, Houts R, Cohen HJ, Corcoran DL, Danese A, Harrington H, Israel S, Levine ME, Schaefer JD, Sugden K, Williams B, Yashin AI, Poulton R, Moffitt TE (2015). Quantification of biological aging in young adults. Proc Natl Acad Sci USA.

[12] Belsky DW, Huffman KM, Pieper CF, Shalev I, Kraus WE (2017). Change in the rate of biological aging in response to caloric restriction: CALERIE Biobank Analysis. J Gerontol Ser A.

[13] Belsky DW, Moffitt TE, Cohen AA, Corcoran DL, Levine ME, Prinz JA, Schaefer J, Sugden K, Williams B, Poulton R, Caspi A (2017). Eleven telomere, epigenetic clock, and biomarker-composite quantifications of biological aging: do they measure the same thing?. Am J Epidemiol.

[14] Bertinato J, Wang KC, Hayward S (2017). Serum magnesium concentrations in the Canadian population and associations with diabetes, glycemic regulation, and insulin resistance. Nutrients.

[15] Biesalski HK, Aggett PJ, Anton R, Bernstein PS, Blumberg J, Heaney RP, Henry J, Nolan JM, Richardson DP, van Ommen B, Witkamp RF, Rijkers GT, Zöllner (2011). 26th Hohenheim Consensus Conference, September 11, 2010 Scientific substantiation of health claims: evidence-based nutrition. Nutrition.

[16] Biesalski HK, Erdman JW, Hathcock J, Ellwood K, Beatty S, Johnson E, Marchioli R, Lauritzen L, Rice HB, Shao A, Griffiths JC (2013). Nutrient reference values for bioactives: new approaches needed? A conference report. Eur J Nutr.

[17] Bird JK, Murphy RA, Ciappio ED, McBurney MI (2017). Risk of deficiency in multiple concurrent micronutrients in children and adults in the United States. Nutrients.

[18] Bischoff-Ferrari HA, Dawson-Hughes B, Staehelin HB, Orav JE, Stuck AE, Theiler R, Wong JB, Egli A, Kiel DP, Henschkowski J (2009). Fall prevention with supplemental and active forms of vitamin D: a meta-analysis of randomised controlled trials. Br Med J.

[19] Bischoff-Ferrari HA, Fischer K, Orav EJ, Dawson-Hughes B, Meyer U, Chocano-Bedoya PO, Meyer OW, Ernst R, Schietzel S, Eberli F, Staehelin HB, Freystätter G, Roas S, Theiler R, Egli A, Wilson NM (2017). Statin use and 25-hydroxyvitamin D blood level response to vitamin D treatment of older adults. J Am Geriatr Soc.

[20] Bou Ghanem EN, Clark S, Du X, Wu D, Camilli A, Leong JM (2015). The alpha-tocopherol form of vitamin E reverses age-associated susceptibility to *Streptococcus pneumoniae* lung infection by modulating pulmonary neutrophil recruitment. J Immunol.

[21] Boucher BJ (2012). The problems of vitamin D insufficiency in older people. Aging Dis.

[22] Centers for Disease Control (2012) 2nd national report on biochemical indicators of diet and nutrition in the US Population. https://www.cdc.gov/nutritionreport/pdf/nutrition_book_complete508_final.pdf. Accessed 13 Jan 2018

[23] Centers for Disease Control (2017) National diabetes statistics report, 2017. Estimates of diabetes and its burden in the United States. https://www.cdc.gov/diabetes/pdfs/data/statistics/national-diabetes-statistics-report.pdf. Accessed 3 Feb 2018

[24] Chen BH, Marioni RE, Colicino E, Peters MJ, Ward-Caviness CK, Tsai PC, Roetker NS, Just AC, Demerath EW, Guan W, Bressler J, Fornage M, Studenski S, Vandiver AR, Moore AZ, Tanaka T, Kiel DP, Liang L, Vokonas P, Schwartz J, Lunetta KL, Murabito JM, Bandinelli S, Hernandez DG, Melzer D, Nalls M, Pilling LC, Price TR, Singleton AB, Gieger C, Holle R, Kretschmer A, Kronenberg F, Kunze S, Linseisen J, Meisinger C, Rathmann W, Waldenberger M, Visscher PM, Shah S, Wray NR, McRae AF, Franco OH, Hofman A, Uitterlinden AG, Absher D, Assimes T, Levine ME, Lu AT, Tsao PS, Hou L, Manson JE, Carty CL, LaCroix AZ, Reiner AP, Spector TD, Feinberg AP, Levy D, Baccarelli A, van Meurs J, Bell JT, Peters A, Deary IJ, Pankow JS, Ferrucci L, Horvath S (2016). DNA methylation-based measures of biological age: meta-analysis predicting time to death. Aging.

[25] Childs BR, Andres BA, Vallier HA (2016). Economic benefit of calcium and vitamin D supplementation: does it outweigh the cost of nonunions?. J Orthop Trauma.

[26] Cianferotti L, Parri S, Gronchi G, Civinini R, Brandi M (2017). The use of cholecalciferol in patients with hip fracture. Clin Cases Miner Bone Metab.

[27] Cieza A, Kamenov K, Officer A, Rosenberg M, Pot AM (2017) Towards operationalizing functional ability. Background paper for the WHO Working Group on Metrics and Research Standards for Healthy Ageing, March 2017 [Also mentioned in Cesari M, Araujo de Carvalho I, Amuthavalli Thiyagarajan J, Cooper C, Martin FC, Reginster JY, Vellas B, Beard JR. (2018) Evidence for the domains supporting the construct of intrinsic capacity. J Gerontol A Biol Sci Med Sci. 10.1093/gerona/gly011 (Epub ahead of print)] 10.1093/gerona/gly011 29408961

[28] Coates JC, Colaiezzi BA, Bell W, Charrondiere UR, Leclercq C (2017). Overcoming dietary assessment challenges in low-income countries: technological solutions. Nutrients.

[29] Cohen AA, Milot E, Yong J, Seplaki CL, Fülöp T, Bandeen-Roche K, Fried LP (2013). A novel statistical approach shows evidence for multi-system physiological dysregulation during aging. Mech Ageing Dev.

[30] Conzade R, Koenig W, Heier M, Schneider A, Grill E, Peters A, Thorand B (2017). Prevalence and predictors of subclinical micronutrient deficiency in German older adults: results from the population-based KORA-Age Study. Nutrients.

[31] Dainelli L, Xu T, Li M, Zimmermann D, Fang H, Wu Y, Detzel P (2017). Cost-effectiveness of milk powder fortified with potassium to decrease blood pressure and prevent cardiovascular events among the adult population in China: a Markov model. BMJ Open [Internet].

[32] de Carvalho IE, Martin FC, Cesari M, Summi Y, Thiyagarajan JA, Beard J (2017) Operationalising the concept of intrinsic capacity in clinical settings. Background paper for the WHO Working Group on Metrics and Research Standards for Healthy Ageing, March 2017 [Also mentioned in Cesari M, Araujo de Carvalho I, Amuthavalli Thiyagarajan J, Cooper C, Martin FC, Reginster JY, Vellas B, Beard JR. (2018) Evidence for the domains supporting the construct of intrinsic capacity. J Gerontol A Biol Sci Med Sci. 10.1093/gerona/gly011 (Epub ahead of print)] 10.1093/gerona/gly011 29408961

[33] de Jongh RT, van Schoor NM, Lips P (2017). Changes in vitamin D endocrinology during aging in adults. Mol Cell Endocrinol.

[34] Del Gobbo LC, Imamura F, Wu JH, de Oliveira Otto MC, Chiuve SE, Mozaffarian D (2013). Circulating and dietary magnesium and risk of cardiovascular disease: a systematic review and meta-analysis of prospective studies. Am J Clin Nutr.

[35] Depp CA, Jeste DV (2006). Definitions and predictors of successful aging: a comprehensive review of larger quantitative studies. Am J Geriatr Psychiatry.

[36] Dibaba DT, Xun P, Song Y, Rosanoff A, Shechter M, He K (2017). The effect of magnesium supplementation on blood pressure in individuals with insulin resistance, prediabetes, or noncommunicable chronic diseases: a meta-analysis of randomized controlled trials. Am J Clin Nutr.

[37] Drewnowski A, Evans WJ (2001). Nutrition, physical activity, and quality of life in older adults summary. J Gerontol Ser A.

[38] Du X, Wang J, Niu X, Smith D, Wu D, Meydani SN (2014). Dietary wolfberry supplementation enhances the protective effect of flu vaccine against influenza challenge in aged mice. J Nutr.

[39] Fontana L, Kennedy BK, Longo VD, Seals D, Melov S (2014). Medical research: treat ageing. Nature.

[40] Frei R, Akdis M, O’Mahony L (2015). Prebiotics, probiotics, synbiotics, and the immune system: experimental data and clinical evidence. Curr Opin Gastroenterol.

[41] Freid VM, Bernstein AB, Bush MA (2012). NCHS Data Brief, Multiple chronic conditions among adults aged 45 and over: trends over the past 10 years. US Centers Dis Control Prev NCHS Data Brief.

[42] Fries JF (2002). Successful aging: an emerging paradigm of gerontology. Clin Geriatr Med.

[43] Girodon F, Lombard M, Galan P, Brunet-Lecomte P, Monget AL, Arnaud J (1997). Effect of micronutrient supplementation on infection in institutionalized elderly subjects: a controlled trial. Ann Nutr Metab.

[44] Gommers LM, Hoenderop JG, Bindels RJ, de Baaij JH (2016). Hypomagnesemia in Type 2 diabetes: a vicious circle?. Diabetes.

[45] Gopinath B, Russell J, Flood VM, Burlutsky G, Mitchell P (2014). Adherence to dietary guidelines positively affects quality of life and functional status of older adults. J Acad Nutr Diet.

[46] Graat JM, Schouten EG, Kok FJ (2002). Effect of daily vitamin E and multivitamin-mineral supplementation on acute respiratory tract infections in elderly persons: a randomized controlled trial. JAMA.

[47] Gregori D, Vecchio MG, Minto C, Zec S, Lamprecht M (2017). The role of fruit and vegetable concentrates in alleviating the growing burden of cardiovascular diseases in USA: evidence from a simulation study. FASEB J.

[48] Han SN, Wu D, Ha WK, Beharka A, Smith DE, Bender BS (2000). Vitamin E supplementation increases T helper 1 cytokine production in old mice infected with influenza virus. Immunology.

[49] Harper S (2014). Economic and social implications of aging societies. Science.

[50] Haslam A, Johnson MA, Hausman DB, Cress ME, Houston DK, Davey A, Poon LW (2014). Georgia Centenarian Study. Vitamin D status is associated with grip strength in centenarians. J Nutr Gerontol Geriatr.

[51] Haslam C, Cruwys T, Haslam SA (2014). “The we’s have it”: evidence for the distinctive benefits of group engagement in enhancing cognitive health in aging. Soc Sci Med.

[52] Hayek MG, Taylor SF, Bender BS, Han SN, Meydani M, Smith DE (1997). Vitamin E supplementation decreases lung virus titers in mice infected with influenza. J Infect Dis.

[53] Healthy Ireland (2015) Healthy Ireland Survey 2015: summary of findings. Dublin, Ireland, http://health.gov.ie/wp-content/uploads/2015/10/Healthy-Ireland-Survey-2015-Summary-of-Findings.pdf. Accessed 10 Aug 2017

[54] Heidenreich PA, Trogdon JG, Khavjou OA, Butler J, Dracup K, Ezekowitz MD (2011). Forecasting the future of cardiovascular disease in the United States. Circulation.

[55] Helpage (2014) Global AgeWatch Index 2014: insight report, summary and methodology. http://www.helpage.org/global-agewatch/rep. Accessed 1 Mar 2018

[56] Hemila H (2016). Vitamin E administration may decrease the incidence of pneumonia in elderly males. Clin Interv Aging.

[57] Holick MF, Binkley NC, Bischoff-Ferrari HA, Gordon CM, Hanley DA, Heaney RP, Murad MH, Weaver CM, Endocrine Society (2011). Evaluation, treatment, and prevention of vitamin D deficiency: an Endocrine Society Clinical Practice Guideline. J Clin Endocrinol Metab.

[58] Institute of Medicine, Food and Nutrition Board (2010) Dietary reference intakes for calcium and vitamin D. National Academy Press, Washington, DC. https://www.ncbi.nlm.nih.gov/books/NBK56070/

[59] Institute of Medicine. Food and Nutrition Board (1998) Dietary reference intakes: thiamin, riboflavin, niacin, vitamin B6, folate, vitamin B12, pantothenic acid, biotin, and choline. National Academy Press, Washington, DC. https://www.ncbi.nlm.nih.gov/books/NBK114310/23193625

[60] IUNA (Irish Universities Nutrition Alliance) (2005) The National Children’s Food Survey (2005). IUNA national child food survey main report. https://www.iuna.net. Accessed 10 Aug 2010

[61] IUNA (Irish Universities Nutrition Alliance) (2008) National Teens’ Food Survey. Main report. https://www.iuna.net. Accessed 10 Aug 2010

[62] IUNA (Irish Universities Nutrition Alliance) (2011) National adult nutrition survey. summary report. https://www.iuna.net. Accessed 10 Aug 2010

[63] IUNA (Irish Universities Nutrition Alliance) (2012) National pre-school nutrition survey. summary report. https://www.iuna.net. Accessed 10 Aug 2010

[64] Jeste DV, Depp CA, Vahia IV (2010). Successful cognitive and emotional aging. World Psychiatry.

[65] Jetté N, Lix LM, Metge CJ, Prior HJ, McChesney J, Leslie WD (2011). Association of antiepileptic drugs with nontraumatic fractures: a population-based analysis. Arch Neurol.

[66] Jetten J, Haslam C, Haslam SA, Dingle G, Jones JM (2014). How groups affect our health and well-being: the path from theory to policy. Soc Issues Policy Rev.

[67] Johnell O, Kanis JA (2006). An estimate of the worldwide prevalence and disability associated with osteoporotic fractures. Osteoporos Int.

[68] Johnson EJ, Vishwanathan R, Johnson MA, Hausman DB, Davey A, Scott TM, Green RC, Miller LS, Gearing M, Woodard J, Nelson PT, Chung HY, Schalch W, Wittwer J, Poon LW (2013). Relationship between serum and brain carotenoids, α-tocopherol, and retinol concentrations and cognitive performance in the oldest old from the Georgia Centenarian Study. J Aging Res.

[69] Johnson MA, Davey A, Hausman DB, Park S, Poon LW (2006). Dietary differences between centenarians residing in communities and in skilled nursing facilities: the Georgia Centenarian Study. Age (Dordr).

[70] Johnson MA, Davey A, Park S, Hausman DB, Poon LW (2008). Georgia Centenarian Study. Age, race and season predict vitamin D status in African American and white octogenarians and centenarians. J Nutr Health Aging.

[71] Johnson MA, Hausman DB, Davey A, Poon LW, Allen RH, Stabler SP (2010). Georgia Centenarian Study. Vitamin B12 deficiency in African American and white octogenarians and centenarians in Georgia. J Nutr Health Aging.

[72] Jung SB, Nagaraja V, Kapur A, Eslick GD (2015). Association between vitamin B12 deficiency and long-term use of acid-lowering agents: a systematic review and meta-analysis. Intern Med J.

[73] Justice J, Miller JD, Newman JC, Hashmi SK, Halter J, Austad SN, Barzilai N, Kirkland JL (2016). Frameworks for proof-of-concept clinical trials of interventions that target fundamental aging processes. J Gerontol A Biol Sci Med Sci.

[74] Jylhävä J, Pedersen NL, Hägg S (2017). Biological age predictors. EBioMedicine.

[75] Kaeberlein M, Rabinovitch PS, Martin GM (2015). Healthy aging: the ultimate preventative medicine. Science.

[76] Kancherla V, Elliott JL, Patel BB, Holland NW, Johnson TM, Khakharia A, Phillips LS, Oakley GP, Vaughan CP (2017). Long term metformin therapy and monitoring for vitamin B12 deficiency among older veterans. J Am Geriatr Soc.

[77] Kanis JA, Johnell O, Oden A, Sembo I, Redlund-Johnell I, Dawson A, De Laet C, Jonsson B (2000). Long-term risk of osteoporotic fracture in Malmo. Osteoporos Int.

[78] Kauppinen A, Paterno JJ, Blasiak J, Salminen A, Kaarniranta K (2016). Inflammation and its role in age-related macular degeneration. Cell Mol Life Sci.

[79] Keller HH, Østbye T, Goy R (2004). Nutritional risk predicts quality of life in elderly community-living Canadians. J Gerontol A Biol Sci Med Sci.

[80] Kennedy BK, Berger SL, Brunet A, Campisi J, Cuervo AM, Epel ES, Franceschi C, Lithgow GJ, Morimoto RI, Pessin JE, Rando TA, Richardson A, Schadt EE, Wyss-Coray T, Sierra F (2014). Geroscience: linking aging to chronic disease. Cell.

[81] Kimmons JE, Blanck HM, Tohill BC, Zhang J, Khan LK (2006). Associations between body mass index and the prevalence of low micronutrient levels among US adults. Med Gen Med.

[82] Klemera P, Doubal S (2006). A new approach to the concept and computation of biological age. Mech Ageing Dev.

[83] Kumar M, Babaei P, Ji B, Nielsen J (2016). Human gut microbiota and healthy aging: recent developments and future prospective. Nutr Healthy Aging.

[84] Lang JE, Anderson L, LoGerfo J, Sharkey J, Belansky E, Bryant L, Prohaska T, Altpeter M, Marshall V, Satariano W, Ivey S, Bayles C, Pluto D, Wilcox S, Goins RT, Byrd RC, Healthy Aging Research Network Writing Group (2006). The prevention research centers healthy aging research network. Prev Chronic Dis.

[85] Langan RC, Goodbred AJ (2017). Vitamin B12 deficiency: recognition and management. Am Fam Physician.

[86] LeDoux MA, Appelhans KR, Braun LA, Dziedziczak D, Liu L, Osiecki H, Wyszumiala E, Griffiths JC (2015). A quality dietary supplement: before you start and after it’s marketed—a conference report. Eur J Nutr.

[87] Lei WT, Shih PC, Liu SJ, Lin CY, Yeh TL (2017). Effect of probiotics and prebiotics on immune response to influenza vaccination in adults: a systematic review and meta-analysis of randomized controlled trials. Nutrients.

[88] Lems WF, Raterman HG (2017). Critical issues and current challenges in osteoporosis and fracture prevention. An overview of unmet needs. Ther Adv Musculoskelet Dis.

[89] Letois F, Mura T, Scali J, Gutierrez L-A, Féart C, Berr C (2016). Nutrition and mortality in the elderly over 10 years of follow-up: the Three-City study. Br J Nutr.

[90] Levine ME (2013). Modeling the rate of senescence: can estimated biological age predict mortality more accurately than chronological age?. J Gerontol A Biol Sci Med Sci.

[91] Lip GYH, Coca A, Kahan T, Boriani G, Manolis AS, Olsen MH, Oto A, Potpara TS, Steffel J, Marín F, de Oliveira Figueiredo MJ, de Simone G, Tzou WS, Chiang CE, Williams B, Dan GA, Gorenek B, Fauchier L, Savelieva I, Hatala R, van Gelder I, Brguljan-Hitij J, Erdine S, Lovic D, Kim YH, Salinas-Arce J, Field M, Reviewers (2017). Hypertension and cardiac arrhythmias: a consensus document from the European Heart Rhythm Association (EHRA) and ESC Council on Hypertension, endorsed by the Heart Rhythm Society (HRS), Asia-Pacific Heart Rhythm Society (APHRS) and Sociedad Latinoamericana de Estimulación Cardíaca y Electrofisiología (SOLEACE). Europace.

[92] Longo VD, Antebi A, Bartke A, Barzilai N, Brown-Borg HM, Caruso C, Curiel TJ, de Cabo R, Franceschi C, Gems D, Ingram DK, Johnson TE, Kennedy BK, Kenyon C, Klein S, Kopchick JJ, Lepperdinger G, Madeo F, Mirisola MG, Mitchell JR, Passarino G, Rudolph KL, Sedivy JM, Shadel GS, Sinclair DA, Spindler SR, Suh Y, Vijg J, Vinciguerra M, Fontana L (2015). Interventions to slow aging in humans: are we ready?. Aging Cell.

[93] López-Otín C, Blasco MA, Partridge L, Serrano M, Kroemer G (2013). The hallmarks of aging. Cell.

[94] Lupton JR, Atkinson SA, Chang N, Fraga CG, Levy J, Messina M, Richardson DP, van Ommen B, Yang Y, Griffiths JC, Hathcock J (2014). Exploring the benefits and challenges of establishing a DRI-like process for bioactives. Eur J Nutr.

[95] Lupton JR, Blumberg JB, L’Abbe M, LeDoux M, Rice HB, von Schacky C, Yaktine A, Griffiths JC (2016). Nutrient reference value: noncommunicable disease endpoints—a conference report. Eur J Nutr.

[96] Maes ML, Fixen DR, Linnebur SA (2017). Adverse effects of proton-pump inhibitor use in older adults: a review of the evidence. Ther Adv Drug Saf.

[97] Melton LJ, Crowson CS, O’Fallon WM (1999). Fracture incidence in Olmsted County, Minnesota: comparison of urban with rural rates and changes in urban rates over time. Osteoporos Int.

[98] Merten C, Ferrari P, Bakker M, Boss A, Hearty A, Leclercq C, Lindtner O, Tlustos C, Verger P, Volatier JL, Arcella D (2011). Methodological characteristics of the national dietary surveys carried out in the European Union as included in the European Food Safety Authority (EFSA) Comprehensive European Food Consumption Database. Food Addit Contam Part A Chem Anal Control Expo Risk Assess.

[99] Meydani SN, Barklund MP, Liu S, Meydani M, Miller RA, Cannon JG (1990). Vitamin E supplementation enhances cell-mediated immunity in healthy elderly subjects. Am J Clin Nutr.

[100] Meydani SN, Barnett JB, Dallal GE, Fine BC, Jacques PF, Leka LS (2007). Serum zinc and pneumonia in nursing home elderly. Am J Clin Nutr.

[101] Meydani SN, Das SK, Pieper CF, Lewis MR, Klein S, Dixit VD (2016). Long-term moderate calorie restriction inhibits inflammation without impairing cell-mediated immunity: a randomized controlled trial in non-obese humans. Aging (Albany NY).

[102] Meydani SN, Endres S, Woods MM, Goldin BR, Soo C, Morrill-Labrode A (1991). Oral (n-3) fatty acid supplementation suppresses cytokine production and lymphocyte proliferation: comparison between young and older women. J Nutr.

[103] Meydani SN, Leka LS, Fine BC, Dallal GE, Keusch GT, Singh MF (2004). Vitamin E and respiratory tract infections in elderly nursing home residents: a randomized controlled trial. JAMA.

[104] Meydani SN, Lichtenstein AH, Cornwall S, Meydani M, Goldin BR, Rasmussen H (1993). Immunologic effects of national cholesterol education panel step-2 diets with and without fish-derived N-3 fatty acid enrichment. J Clin Invest.

[105] Meydani SN, Meydani M, Blumberg JB, Leka LS, Siber G, Loszewski R (1997). Vitamin E supplementation and in vivo immune response in healthy elderly subjects. A randomized controlled trial. JAMA.

[106] Meydani SN, Ribaya-Mercado JD, Russell RM, Sahyoun N, Morrow FD, Gershoff SN (1991). Vitamin B-6 deficiency impairs interleukin 2 production and lymphocyte proliferation in elderly adults. Am J Clin Nutr.

[107] Michel JP, Sadana R (2017). Healthy ageing concepts and measures. JAMDA.

[108] Moffitt TE, Belsky DW, Danese A, Poulton R, Caspi A (2016). The longitudinal study of aging in human young adults: knowledge gaps and research agenda. J Gerontol A Biol Sci Med Sci.

[109] Montoliu I, Scherer M, Beguelin F, DaSilva L, Mari D, Salvioli S, Martin FP, Capri M, Bucci L, Ostan R, Garagnani P, Monti D, Biagi E, Brigidi P, Kussmann M, Rezzi S, Franceschi C, Collino S (2014). Serum profiling of healthy aging identifies phospho- and sphingolipid species as markers of human longevity. Aging.

[110] Moskalev A, Chernyagina E, Tsvetkov V, Fedintsev A, Shaposhnikov M, Krut’ko V, Zhavoronkov A, Kennedy BK (2016). Developing criteria for evaluation of geroprotectors as a key stage toward translation to the clinic. Aging Cell.

[111] Nakagawa T, Cho J, Gondo Y, Martin P, Johnson MA, Poon LW, Hirose N (2017). Subjective well-being in centenarians: a comparison of Japan and the United States. Aging Ment Health.

[112] NASEM (2017). The National Academies of Sciences, Engineering, and Medicine; Health and Medicine Division; Food and Nutrition Board; Food Forum. Nutrition Across the Lifespan for Healthy Aging: Proceedings of a Workshop.

[113] Newgard CB, Sharpless NE (2013). Coming of age: molecular drivers of aging and therapeutic opportunities. J Clin Invest.

[114] Newman JC, Milman S, Hashmi SK, Austad SN, Kirkland JL, Halter JB, Barzilai N (2016). Strategies and challenges in clinical trials targeting human aging. J Gerontol Ser A.

[115] Nikolich-Zugich J, Messaoudi I (2005). Mice and flies and monkeys too: caloric restriction rejuvenates the aging immune system of non-human primates. Exp Gerontol.

[116] O’Mahony C, Vilone G (2013). Compiled European Food Consumption Database. Support Publ.

[117] ODIN (2018) http://www.odin-vitd.eu/. Accessed 3 Jan 2018

[118] ODS (2018) Office of Dietary Supplements B12 Fact Sheet for health care professionals. https://ods.od.nih.gov/factsheets/VitaminB12-HealthProfessional/. Accessed 11 Mar 2018

[119] Olivieri F, Capri M, Bonafè M, Morsiani C, Jung HJ, Spazzafumo L, Viña J, Suh Y (2017). miRNAs and miRNA shuttles as biomarkers: perspective trajectories of healthy and unhealthy aging. Mech Ageing Dev.

[120] Out M, Kooy A, Lehert P, Schalkwijk CA, Stehouwer CDA (2018). Long-term treatment with metformin in type 2 diabetes and methylmalonic acid: post hoc analysis of a randomized controlled 4.3 year trial. J Diabetes Complicat.

[121] Passeri G, Pini G, Troiano L, Vescovini R, Sansoni P, Passeri M, Gueresi P, Delsignore R, Pedrazzoni M, Franceschi C (2003). Low vitamin D status, high bone turnover, and bone fractures in centenarians. J Clin Endocrinol Metab.

[122] Peel NM, McClure RJ, Bartlett HP (2005). Behavioral determinants of healthy aging. Am J Prev Med.

[123] Pigat S, Connolly A, Cushen M, Cullen M, O’Mahony C (2018). A probabilistic intake model to estimate the impact of reformulation by the food industry among Irish consumers. Int J Food Sci Nutr.

[124] Pigat S, Kiely M (2017). Assessing vitamin D safety following fortification and supplementation intake scenarios using the EFSA Comprehensive Database: assessing vitamin D safety following fortification and supplementation ODIN approach. Proc Nutr Soc.

[125] Plumb J, Pigat S, Bompola F, Cushen M, Pinchen H, Nørby E, Astley S, Lyons J, Kiely M, Finglas P (2017) eBASIS (Bioactive Substances in Food Information Systems) and bioactive intakes: major updates of the bioactive compound composition and beneficial bioeffects database and the development of a probabilistic model to assess intakes in Europe. Nutrients [Internet]. 9:320. http://www.mdpi.com/2072-6643/9/4/320. Accessed 1 Mar 2018 10.3390/nu9040320 PMC5409659 28333085

[126] Poon LW, Jazwinski M, Green RC, Woodard JL, Martin P, Rodgers WL, Johnson MA, Hausman D, Arnold J, Davey A, Batzer MA, Markesbery WR, Gearing M, Siegler IC, Reynolds S, Dai J (2007). Methodological considerations in studying centenarians: lessons learned from the Georgia Centenarian Studies. Annu Rev Gerontol Geriatr.

[127] Prasad AS, Beck FW, Bao B, Fitzgerald JT, Snell DC, Steinberg JD (2007). Zinc supplementation decreases incidence of infections in the elderly: effect of zinc on generation of cytokines and oxidative stress. Am J Clin Nutr.

[128] Ren Z, Na L, Xu Y, Rozati M, Wang J, Xu J (2012). Dietary supplementation with lacto-wolfberry enhances the immune response and reduces pathogenesis to influenza infection in mice. J Nutr.

[129] Rocca WA, Petersen RC, Knopman DS, Hebert LE, Evans DA, Hall KS, Gao S, Unverzagt FW, Langa KM, Larson EB, White LR (2011). Trends in the incidence and prevalence of Alzheimer’s disease, dementia, and cognitive impairment in the United States. Alzheimer Dement.

[130] Rosanoff A, Weaver CM, Rude RK (2012). Suboptimal magnesium status in the United States: are the health consequences underestimated?. Nutr Rev.

[131] Rose MR, Flatt T, Graves JL, Greer LF, Martínez DE, Matos M, Mueller LD, Shmookler Reis RJ, Shahrestani P (2012) A new definition of aging? Front Genet (3):134 10.3389/fgene.2012.00134 PMC3400891 22833755

[132] Rosique-Esteban N, Guasch-Ferré M, Hernández-Alonso P, Salas-Salvadó J (2018). Dietary magnesium and cardiovascular disease: a review with emphasis in epidemiological studies. Nutrients.

[133] Rowe JW, Kahn RL (1997). Successful aging. Gerontologist.

[134] Sadana R, Posarac A (2018). Need, use of services and relationship with intrinsic capacity, Department of Ageing and Life Course.

[135] Sadana R, Blas E, Budhwani S, Koller T, Paraje G (2016). Healthy ageing: raising awareness of inequalities, determinants, and what can be done to improve health equity. Gerontologist.

[136] Shao A, Drewnowski A, Willcox DC, Krämer L, Lausted C, Eggersdorfer M, Mathers J, Bell JD, Randolph RK, Witkamp R, Griffiths JC (2017). Optimal nutrition and the ever-changing dietary landscape: a conference report. Eur Jrnl Nutrit.

[137] Simental-Mendia LE, Sahebkar A, Rodriguez-Moran M, Zambrano-Galvan G, Guerrero-Romero F (2017). Effect of magnesium supplementation on plasma C-reactive protein concentrations: a systematic review and meta-analysis of randomized controlled trials. Curr Pharm Des.

[138] Somogyi A, Hathcock J, Biesalski HK, Blumberg JB, Antoine JM, Edwards G, Prock P (2011). Scientific issues related to Codex Alimentarius goals: a review of principles with examples. Reg Toxicol Pharmacol.

[139] Smith MR, Micha R, Golden CD, Mozaffarian D, Myers SS (2016). Global Expanded Nutrient Supply (GENuS) Model: a new method for estimating the global dietary supply of nutrients. PLoS One.

[140] Stepler R. Pew Research C (2016) World’s centenarian population projected to grow eightfold by 2050. http://www.pewresearch.org/fact-tank/2016/04/21/worlds-centenarian-population-projected-to-grow-eightfold-by-2050/. Accessed 15 Feb 2018

[141] Tinetti ME (2003). Clinical practice. Preventing falls in elderly persons. N Engl J Med.

[142] Tricco AC, Thomas SM, Veroniki AA, Hamid JS, Cogo E, Strifler L, Khan PA, Robson R, Sibley KM, MacDonald H, Riva JJ, Thavorn K, Wilson C, Holroyd-Leduc J, Kerr GD, Feldman F, Majumdar SR, Jaglal SB, Hui W, Straus SE (2017). Comparisons of interventions for preventing falls in older adults: a systematic review and meta-analysis. JAMA.

[143] Tyrovolas S, Haro JM, Mariolis A, Piscopo S, Valacchi G, Tsakountakis N, Zeimbekis A, Tyrovola D, Bountziouka V, Gotsis E, Metallinos G, Tur JA, Matalas AL, Lionis C, Polychronopoulos E, Panagiotakos D (2014). Successful aging, dietary habits and health status of elderly individuals: a k-dimensional approach within the multi-national MEDIS study. Exp Gerontol.

[144] United Nations DESA. (2013) Department of Economic and Social Affairs, Population Division. World Population Ageing 2013. http://www.un.org/en/development/desa/population/publications/pdf/ageing/WorldPopulationAgeing2013.pdf. Accessed 1 Mar 2018

[145] Vidal K, Benyacoub J, Sanchez-Garcia J, Foata F, Segura-Roggero I, Serrant P (2010). Intake of a milk-based wolfberry formulation enhances the immune response of young-adult and aged mice. Rejuvenation Res.

[146] Vidal K, Bucheli P, Gao Q, Moulin J, Shen LS, Wang J (2012). Immunomodulatory effects of dietary supplementation with a milk-based wolfberry formulation in healthy elderly: a randomized, double-blind, placebo-controlled trial. Rejuvenation Res.

[147] Wayne SJ, Baumgartner K, Baumgartner RN, Bernstein L, Bowen DJ, Ballard-Barbash R (2006). Diet quality is directly associated with quality of life in breast cancer survivors. Breast Cancer Res Treat.

[148] Weaver CM, Alexander DD, Boushey CJ, Dawson-Hughes B, Lappe JM, LeBoff MS, Liu S, Looker AC, Wallace TC, Wang DD (2016). Calcium plus vitamin D supplementation and risk of fractures: an updated meta-analysis from the National Osteoporosis Foundation. Osteoporosis Int.

[149] Weindruch R, Gottesman SR, Walford RL (1982). Modification of age-related immune decline in mice dietarily restricted from or after mid-adulthood. Proc Natl Acad Sci U S A.

[150] West LA, Goodkine D, He W (2014) 65 + in the United States: 2010, current population report. In: Bureau USC, editor. pp. 23–212. http://www.census.gov/content/dam/Census/library/publications/2014/demo/p23-212.pdf. Accessed 15 May 2018

[151] WHO (2001) The International Classification of Functioning, Disability and Health; World Health Organization. http://www.who.int/classifications/icf/en/. Accessed 1 Mar 2018

[152] WHO (2002) Towards a common language for functioning, disability and health. http://www.who.int/classifications/icf/icfbeginnersguide.pdf. Accessed 30 Mar 2018

[153] WHO (2015) What is healthy ageing? http://www.who.int/ageing/healthy-ageing/en/. Accessed 30 Mar 2018

[154] WHO (2015) Word report on ageing and health. http://www.who.int/ageing/events/world-report-2015-launch/en/. Accessed 15 May 2018

[155] WHO (2016) The Global strategy and action plan on ageing and health http://www.who.int/ageing/global-strategy/en/. Accessed 30 Mar 2018

[156] WHO (2017) Diabetes fact sheet. Updated November http://www.who.int/mediacentre/factsheets/fs312/en/. Accessed 3 Jan 2018

[157] WHO (2018) Nutrition for older persons. http://www.who.int/nutrition/topics/ageing/en/index1.html. Accessed 3 Jan 2018

[158] World Economic Forum (2009) Transforming pensions and healthcare in a rapidly ageing world: opportunities and collaborative strategies. https://www.weforum.org/reports/transforming-pensions-and-healthcare-rapidly-ageing-world-opportunities-and-collaborative-strategies. Accessed 1 Mar 2018

[159] Wu D, Han SN, Meydani M, Meydani SN (2006). Effect of concomitant consumption of fish oil and vitamin E on T cell mediated function in the elderly: a randomized double-blind trial. J Am Coll Nutr.

[160] Wysokiński A, Sobów T, Kłoszewska I, Kostka T (2015). Mechanisms of the anorexia of aging: a review. Age.

[161] Ziaaldini MM, Marzetti E, Picca A, Murlasits Z (2017). Biochemical pathways of sarcopenia and their modulation by physical exercise: a narrative review. Front Med.

[162] Zinger A, Cho WC, Ben-Yehuda A (2017). Cancer and aging—the inflammatory connection. Aging Dis.

